# Genomic insights into host-associated variants and transmission features of a ToBRFV isolate from Mexico

**DOI:** 10.3389/fpls.2025.1580000

**Published:** 2025-08-15

**Authors:** Erika Janet Zamora-Macorra, Daniel Leobardo Ochoa-Martínez, Claudia Yaritza Chavarín-Camacho, Rosemarie W. Hammond, Katia Aviña-Padilla

**Affiliations:** ^1^ Invernadero de virus fitopatógenos. Universidad Autónoma Chapingo, Preparatoria Agrícola, Texcoco, Mexico; ^2^ Laboratorio de virus fitopatógenos, Colegio de Postgraduados, Fitopatología, Texcoco, Mexico; ^3^ Molecular Plant Pathology Laboratory, United States Department of Agriculture (USDA), Agricultural Research Service, Beltsville Agricultural Research Center, Beltsville, MD, United States; ^4^ Laboratorio de Bioinformática y Redes Complejas, Centro de Investigación y de Estudios Avanzados del Instituto Politécnico Nacional (IPN), Departamento de Ingeniería Genética, Unidad Irapuato, Irapuato, Guanajuato, Mexico

**Keywords:** ToBRFV, genomic variability, host-specific adaptation, seed-borne transmission, tomato, single nucleotide variants, SNVs, agricultural biosecurity

## Abstract

Tomato brown rugose fruit virus (ToBRFV) poses a global threat to tomato and pepper production due to its high transmissibility and adaptability. Understanding its genomic features and transmission mechanisms is critical for effective disease management. We characterized the genome and biological properties of a ToBRFV isolate from Mexico. Comparative genomic analyses were conducted using 100 global genomes, with particular focus on single nucleotide variants (SNVs) and their distribution across different host species. Phylogenetic analysis and experimental bioassays, including seed transmission tests, were also performed. Phylogenetic analysis revealed genetic proximity between the Mexican isolate and strains from Mexico, USA, Canada, Israel, and China, indicating shared transmission routes. Genomic comparisons confirmed general sequence stability, but SNVs were found in the 126-kDa replicase, particularly within the methyltransferase domain. These SNVs exhibited host-associated patterns, with conserved profiles in tomato and unique substitutions in *Capsicum annum*, *Solanum nigrum*, and *Citrullus lanatus*. Bioassays demonstrated susceptibility in additional solanaceous hosts, and seed transmission assays in *Nicotiana rustica* indicated reduced germination and low-frequency viral detection in seedlings. The study highlights the genomic conservation and host-specific variation in ToBRFV, suggesting that the methyltransferase domain may undergo differential evolutionary pressures. The findings provide valuable insights for improving risk assessment, seed health testing, and biosecurity measures.

## 1 Introduction

Tomato brown rugose fruit virus (ToBRFV), an emergent tobamovirus, poses a critical threat to global agriculture, particularly affecting essential Solanaceae crops like tomato (*Solanum lycopersicum*) and pepper (*Capsicum annuum*) (Fidan et al., 2020; Magaña-Álvarez et al., 2021). This rod-shaped, positive-sense single-stranded RNA virus (+ssRNA) is notable for its robustness and high transmissibility, with rapid spread reported across key agricultural regions (Luria et al., 2017). In greenhouse settings, ToBRFV transmission is predominantly mechanical, facilitated by routine agricultural practices that inadvertently promote viral spread (Panno et al., 2020).

The genome of ToBRFV consists of approximately 6,400 nucleotides organized into four open reading frames (ORFs) (Salem et al., 2023). This genomic architecture is central to ToBRFV’s infectivity, adaptability, and spread among plant hosts. The viral replication machinery is formed by the proteins encoded in ORF1 and ORF2, namely the 126 kDa and 183 kDa replicase-associated proteins. ORF1 directly encodes the 126-kDa protein (p126), which includes methyltransferase and helicase domains necessary for viral RNA synthesis and host immune suppression, as noted in related tobamoviruses (Ishibashi and Ishikawa, 2016). A readthrough mechanism of the stop codon between ORF1 and ORF2 produces the 183-kDa protein (p183), containing a C-terminal RNA-dependent RNA polymerase (RdRp) domain essential for viral replication and transcription (Ishibashi and Ishikawa, 2016. The RdRp domain plays a central role in the life cycle of positive-sense RNA viruses by catalyzing the synthesis of complementary negative-sense RNA strands, which serve as templates for new genomic and subgenomic RNAs. This process ensures the replication of the viral genome and production of viral proteins required for infection and spread. Moreover, the inherent low fidelity of RdRp and its interactions with host factors influence viral mutation rates and adaptability (Ahlquist, 2006; te Velthuis, 2014; Nagy and Pogany, 2011; de Farias et al., 2017). ToBRFV’s systemic infection capacity is driven by ORF3 and ORF4, which are translated from subgenomic RNAs (sgRNA1 and sgRNA2, respectively). ORF3 encodes a 30-kDa movement protein (MP) that enables cell-to-cell viral spread by modifying plasmodesmata, ensuring effective systemic infection. ORF4 encodes the 17.5-kDa coat protein (CP), which encapsulates the viral RNA, thereby enhancing stability, protecting against degradation, and facilitating both intracellular and inter-host transmission (Salem et al., 2016). The 5′ untranslated region (UTR), rich in CAA repeats, and the 3′ UTR with tRNA-like structures further support ToBRFV’s resilience and adaptability across different host plants.

Genomic and evolutionary analyses are essential for understanding the transmission pathways and host adaptations of the virus. These studies enable researchers to identify adaptive mutations, trace transmission routes, and infer evolutionary relationships that facilitate the virus’s establishment across diverse hosts and environmental conditions (Rizzo et al., 2021; Abrahamian et al., 2022; Zisi et al., 2024). Such information is critical in designing targeted containment strategies tailored to the unique genetic characteristics of local ToBRFV strains.

Recent genomic studies reveal that ToBRFV exhibits remarkably low variability, supporting a monophyletic origin, with over 99% nucleotide identity shared across all known genomic sequences (Abrahamian et al., 2022; Çelik et al., 2022). Limited single-nucleotide variants (SNVs) have been identified, showing a relatively even distribution across the genome without distinct hotspots of variation (Van de Vossenberg et al., 2021). This high sequence conservation and limited variability underscore ToBRFV’s rapid global spread from a single origin, likely driven by international trade in tomato seeds and fruits prior to the implementation of stringent control measures. A recent observation in the Netherlands highlights the emergence of a novel phylogenetic clade, potentially resulting from the unauthorized use of an isolate for cross-protection (Van de Vossenberg et al., 2021). This finding underscores the importance of strict adherence to quarantine regulations to effectively manage this pathogen and prevent further spread (Salem et al., 2023; Botermans et al., 2023).

ToBRFV has rapidly become a focus of agricultural biosecurity efforts worldwide due to its resilience across diverse environments (Datab https://gd.eppo.int/taxon/TOBRFV/distributions). Mexico, with its rich biodiversity in Solanaceae species, including endemic varieties, has emerged as a hotspot for ToBRFV transmission (Vargas-Mejía et al., 2023; García-Estrada et al., 2022). The country’s unique agricultural and ecological dynamics make it a critical region for studying ToBRFV’s adaptation and transmission. Since its detection in Mexico in 2018 (Rodríguez-Mendoza et al., 2019), ToBRFV has established itself across all major tomato-growing regions, raising concerns about its ability to overcome resistance genes (*Tm-1, Tm-2*, and *Tm-22*) traditionally used in tomato cultivars, leading to significant crop losses in high-density greenhouse systems (Salem et al., 2023). A recent second introduction of ToBRFV into Mexico, related to strains from the Netherlands and the Middle East, underscores the virus’s global transmission risk. Seed coat and epicotyl Reverse Transcription Polymerase Chain Reaction (RT-PCR) testing revealed a 9% transmission rate, highlighting the role of seeds in viral dissemination (Vargas-Mejía et al., 2023).

Although tomato and pepper are ToBRFV’s primary natural hosts, experimental inoculations reveal the virus’s ability to infect a broad range of species, including *Chenopodium* and *Nicotiana* spp., demonstrating remarkable adaptability. A key factor in this adaptability is ToBRFV’s genomic plasticity, which allows host-specific mutations that enhance viral fitness in economically significant hosts such as tomato and pepper, as well as in endemic species like *Solanum nigrum* (Salem et al., 2022), *Solanum eleagnifolium*, *Solanum rostratum* (Matzrafi et al., 2023), *Ipomoea purpurea, Mirabilis jalapa*, and *Clematis drummondii* (Vasquez-Gutierrez et al., 2024). Host-driven mutations enhance ToBRFV’s infectivity and replication, enabling its persistence in cultivated and wild plants. This adaptability highlights the need for targeted management strategies, especially in biodiverse regions like Mexico, where endemic species may serve as reservoirs.

ToBRFV spreads mechanically and through seeds, where it primarily resides on the seed coat and endosperm, without affecting the embryo. Although seed-to-seedling transmission rates are relatively low (0.08% to 1.8%), the virus remains a biosecurity concern, especially when micro-wounds in seedlings facilitate infection (Salem et al., 2022; Davino et al., 2020). Seed-borne transmission enables ToBRFV to evade containment, facilitating cross-border spread and threatening global trade, underscoring the need for strict seed surveillance and treatment protocols.

Herein we provide a detailed characterization of ToBRFV isolate from Mexico, emphasizing host-specific mutations, adaptive genetic traits, and seed-borne transmission potential. By integrating genomic analysis, experimental validation, and biosecurity considerations, this research advances understanding of ToBRFV’s adaptability and transmission dynamics, addressing both regional and global challenges posed by this significant agricultural pathogen.

## 2 Materials and methods

### 2.1 Location and field sampling

In September 2020, tomato plants *(S. lycopersicum*) exhibiting classic symptoms of ToBRFV infection - including chlorosis, leaf narrowing, and mosaics - were sourced from commercial greenhouses in Colima, Mexico. Subsequent RT-PCR testing confirmed the presence of ToBRFV in the collected samples, primers ToBRFV-FMX (AACCAGAGTCTTCCTATACTCGGAA) and ToBRFV-RMX (CTCWCCATCTCTTAATAATCTCCT) and protocols were adapted from Rodríguez-Mendoza et al. (2019), (**Supplementary Figure S1**).

### 2.2 Genome sequencing and analysis workflow

RNA extraction was performed using CTAB2%-Trizol^®^ following the protocol described by Jordon-Thaden et al. (2015) (with minor modifications), followed by library preparation involving fragmentation and adapter ligation. The libraries were amplified, purified, and sequenced using Illumina’s Sequencing by Synthesis (SBS) technology by Psomagen^®^, Rockville Maryland USA. Raw sequencing data in BCL format was converted to FASTQ files for downstream analysis. Quality control on the raw reads included assessments of total bases and GC content, along with trimming to remove adapter sequences. The cleaned reads were assembled *de novo* using SPADES, with parameters optimized for contig number and N50. Assembly accuracy was validated by mapping the reads back to the genome, ensuring precise genomic reconstruction. Functional annotation was performed using the conserved domains tool (https://www.ncbi.nlm.nih.gov/Structure/cdd/wrpsb.cgi) (Wang et al., 2023; Lu et al., 2020; Marchler-Bauer et al., 2017). Then, we implemented a bioinformatics workflow illustrated in **Supplementary Figure S2**, accessible at https://github.com/kap8416/TOBRFV-Genome-Analyses/tree/main. The full sequences of 100 ToBRFV genomes were selected based on their closeness phylogenetic relationship with our isolate and retrieved from the GenBank database (https://www.ncbi.nlm.nih.gov/genbank/, accessed in July 2024). Metadata, including geographical location and host information, was extracted and analyzed using custom scripts in the RStudio environment (https://www.rstudio.com/). A total of 101 ToBRFV genome sequences—including 100 publicly available genomes and our newly sequenced isolate—were used to conduct subsequent bioinformatic analyses.

### 2.3 Phylogenetic analysis

ToBRFV genome sequences were aligned using the ClustalW algorithm. All input sequences were provided in FASTA format. This alignment was then used as input for phylogenetic analysis in IQ-TREE2 using bash scripts. The analysis employed the Maximum Likelihood (ML) method, with ModelFinder automatically selecting the best-fitting substitution model. To evaluate clade support, 1000 ultrafast bootstrap replicates were applied. The resulting phylogenetic tree was visualized in Rstudio using the ggtree package for enhanced visualization. A pairwise sequence identity matrix was generated using the ape package to quantify nucleotide identity percentages between isolates and visualized as a heatmap with the pheatmap package. Then, a Neighbor-Net phylogenetic network was generated using the phangorn and ape packages to examine relationships among the ToBRFV isolates. Finally, key genetic diversity parameters, including haplotype diversity (Hd), the number of haplotypes (H), nucleotide diversity (π), and the average nucleotide differences (k), were calculated with the pegas and ape packages. A radar plot was created with the fmsb package to visually compare these diversity indices across isolates.

### 2.4 Genomic variation and distribution analysis of the ToBRFV isolate from Mexico

#### 2.4.1 Nucleotide variation analysis

To detect specific genomic differences between the ToBRFV isolate from Mexico (NCBI accession: BankIt2895689 Mexican PQ628197) and the publicly available ToBRFV genomes, a nucleotide variation analysis was performed using the Biostrings package in RStudio. Alignment with reference genomes enabled identification of nucleotide positions where the Mexican isolate diverged. To ensure consistent variability measurement, observed differences at each position were normalized for sequence length and alignment quality. Statistical summaries, including mean and standard deviation, were computed to highlight regions with higher or lower mutation rates. Visualization of normalized difference frequencies across the genome was achieved using ggplot2, with elevated values pinpointing sites of increased variability. To analyze sequence variability and complexity in the ToBRFV isolate from Mexico, Shannon entropy was calculated for each nucleotide position based on alignment data. Entropy values were plotted alongside genomic differences to identify regions of high diversity and potential functional significance. Sliding window analyses smoothed trends, and correlation metrics quantified the relationship between entropy and mutation frequency. These approaches provided insights into sequence diversity and its genomic distribution. Correlation metrics, including Pearson and Spearman coefficients, quantified the association between mutation frequency and entropy. Data was analyzed and visualized using custom scripts in RStudio.

#### 2.4.2 Geographical distribution of ToBRFV isolates

Geographical metadata associated with each ToBRFV isolate was extracted and visualized using ggplot2 (3.3.2) and sf (0.9-6) packages in RStudio. A world map annotated with counts of genetically similar isolates from each region provided a visual overview of the global distribution and regional clustering of ToBRFV isolates closely related to the ToBRFV genome from Mexico, illustrating the global dissemination patterns of the virus.

#### 2.4.3 Alignment scores by host and geographical distribution

To examine alignment scores in relation to natural hosts and geographical distribution, alignment scores for each ToBRFV genome sequence were calculated using the msa package (version 1.22.0). These scores were combined with host and geographic data to create an integrated dataset. A scatter plot was generated with ggplot2 illustrating alignment score variations across hosts and geographical locations.

#### 2.4.4 Host-specific nucleotide and amino acid differences

To explore single nucleotide variation (SNVs) associated with different hosts, sequence variants were identified across host groups using the Biostrings package. These differences were integrated with host data creating a dataset linking genetic variation to host information. The nucleotide sequence of the ToBRFV Mexican isolate was preprocessed to ensure continuity for analysis. Codon and amino acid translations were performed using a custom R script with the seqinr package to map nucleotide positions to codons and amino acids. The methyltransferase (280–1365 nt) and helicase (2543–3315 nt) domains, critical for replication and transcription, were analyzed to evaluate the impact of SNVs. Mutation effects were categorized as *“Change”* or *“No change”* based on amino acid substitutions. Using ggplot2, scatterplots visualized SNVs. The structural and functional impact of the substitution in the methyltransferase domain was assessed using Swiss-Model (https://swissmodel.expasy.org) for structure prediction, DynaMut2 (https://biosig.lab.uq.edu.au/dynamut2/)for stability analysis (ΔΔG), the I-Mutant2.0 tool (https://folding.biofold.org/i-mutant/i-mutant2.0.html) for thermodynamic prediction, and the PROVEAN (http://provean.jcvi.org/index.php) to evaluate the variant’s potential biological effect.

### 2.5 Experimental validation of ToBRFV hosts

#### 2.5.1 Inoculum preparation, RNA extraction and RT-PCR confirmation

To prepare the inoculum, 1 g of ToBRFV-infected tomato leaves was macerated in 10 mL of phosphate buffer (pH 7.0). The resulting homogenate was mechanically inoculated onto the leaves of *Nicotiana glutinosa* plants (n = 3) using a cotton swab and carborundum to facilitate viral entry through mechanical abrasion. Local necrotic lesions developed approximately six days post-inoculation dpi. This infection was sequentially transferred through four generations of *N. glutinosa* plants to ensure consistency. The final inoculum was then used to infect tomato plants. This inoculum was used for further host bioassays and transmission studies. Plant tissues were first mechanically macerated in phosphate buffer (0.1 M potassium phosphate, pH 7.0; 0.1 M sodium sulfite; 1% β-mercaptoethanol) and used to inoculate uninfected tomato seedlings which were confirmed to be infected via conventional RT-PCR at 30 (dpi). Infected plant tissues (100 mg of foliar tissue including pedicels, leaves, and shoots) were processed for total RNA extraction using PlantRNAeasy miniKit^®^, following the manufacturer’s instructions. Then the ToBRFV presence was confirmed by RT-PCR, with specific primers ToBRFV-FMX (AACCAGAGTCTTCCTATACTCGGAA) and ToBRFV-RMX (CTCWCCATCTCTTAATAATCTCCT) reported by Rodríguez-Mendoza et al., 2019 and following an adapted version of their protocol as previously recently reported in Zamora-Macorra (2023). Resulting amplicons were sequenced to verify viral identity.

#### 2.5.2 Host bioassays

To identify potential ToBRFV hosts, mechanical inoculations were performed on five biological replicates from each of the following species; a) Solanaceae family: tomato *(S. lycopersicum L.)*, tomatillo *(P. ixocarpa)*, tobacco *(N. rustica)*, pepper *(C. annuum)*, eggplant *(S. melongena L.)*; b) Cucurbitaceae family: watermelon *(Citrullus lanatus)*, cantaloupe (*Cucumis melo L.)*, squash (*Cucurbita pepo*), and cucumber (*Cucumis sativus L.*). To prepare the inoculum source, 1 g of infected plant material, obtained in section 2.5.1, was macerated in 10 mL of phosphate buffer (0.1 M potassium phosphate, pH 7.0; 0.1 M sodium sulfite; 1% β-mercaptoethanol). This homogenate was mechanically inoculated by rubbing it onto one cotyledon and one true leaf using a swab, after applying carborundum as an abrasive to facilitate infection. Additionally, three plants from each species were mock inoculated as controls. The plants were maintained under greenhouse conditions and observed daily to record symptoms. After 30 dpi, plants were tested for virus presence using nested RT-PCR with specific primers TobN up3 (GGCGYTGCARACIATHGTITAYCA), TobN do4 (GTRTTICCIATRAAIGTIGTIACRTC) and TobN do4G (GCCGATRAAGGTGGTGACRTC) described by Dovas et al. (2004), and by employing ELISA using antibodies against TMV (Agdia^®^), which exhibit cross-reactivity. ELISA was performed following the manufacturer’s instructions provided by Agdia^®^. This experiment was performed thrice in exposed to natural environmental conditions. The observed differences in temperature and light exposure across repetitions reflect seasonal variation, as the greenhouse facility operates under passive conditions without artificial climate regulation. First in October 2019, second in February 2020, and last in April 2020. During the first experiment, the average monthly temperature in the greenhouse ranged from 13°C to 23°C with a 12-hour light:12 hours dark cycle. During the second experiment, the temperature went from 11° to 26° with an 11-hour light:13 hours dark cycle. The temperature during the third experiment ranged from 14° to 29°C with a 13-hour light:11 hours dark cycle.

### 2.6 Seed Transmission test: acquisition of seeds

For seed transmission test, thirty healthy *N. rustica* tobacco plants at the two true leaves stage were subjected to mechanical inoculation with the virus as described in 2.5.2. The selection of *N. rustica* was based on its comparatively lower symptom severity upon infection, which did not compromise plant fertility. This allowed for consistent capsule and seed production, a critical factor for evaluating vertical transmission of the virus. Simultaneously, a separate group of plants was mock inoculated with a homogenate of healthy tomato tissue and used as controls. Following inoculation, all plants were cultivated under stringent greenhouse conditions, with a stable 12-hour light:12-hour dark photoperiod, and temperatures maintained between 16°C and 24°C. As the plants reached fruiting, seeds were carefully harvested from each plant once their capsules opened. These seeds were then stored at a cool 4°C for preservation. To validate the successful transmission and presence of ToBRFV in the tobacco plants, a post-harvest assessment was carried out. This process involved the extraction of total RNA from pedicels, leaves, and shoots, followed by nested RT-PCR amplification using primer sets based on the protocol established by Dovas et al. (2004).

#### 2.6.1 Seed germination test

Embryo-less seeds were carefully identified under a stereoscopic microscope and discarded. The remaining seeds were divided into three distinct seed lots, each comprising 100 seeds obtained from *N. rustica* plants previously infected with ToBRFV, as well as from mock-inoculated control plants. For germination assays, each seed lot was placed in a sterile Petri dish lined with moistened filter paper using sterile distilled water. To promote germination, an initial incubation phase was performed under continuous light at 23°C for 72 hours, a condition commonly used to synchronize germination and activate photoreceptors known to influence seed dormancy release. This was followed by a temperature fluctuation regime of 18°C (dark period) and 24°C (light period) under a 12 h light/12 h dark photoperiod. These light and temperature settings were selected based on previous studies reporting optimal germination parameters for tobacco species and related solanaceous crops under controlled environments (Florentine et al., 2014; Mukarati et al., 2013; Liu et al., 2023). After 20 days, germinated seedlings in each dish were counted. All germination experiments were performed in triplicate to ensure reproducibility and statistical robustness.

#### 2.6.2 Estimation of percentage of infection in seeds or seedlings and experimental method to determine percentage of infected seeds

To determine the infection rate of ToBRFV in *N. rustica* seeds and seedlings, we used Albrechtsen’s (2006) maximum likelihood estimation method, described in **Supplementary Figure S3**. Samples were divided into subgroups (N), each with a set number of specimens (n), and the infection percentage was calculated using the formula P=[1−(Y/N)^1/n^]×100, where Y is the number of infection-negative subgroups. This method was applied to *N. rustica* seedlings grown from seeds of ToBRFV-infected plants. To assess ToBRFV infection in seedlings, a total of 2,250 total (15 subsets of 250 seeds) collected from ToBRFV- infected tobacco plants were germinated under the conditions described in Section 2.6.3. After 20 days of growth under controlled conditions, the seedlings were harvested, counted, and immediately stored at –20 °C to preserve RNA integrity. This 20-day incubation period allowed for potential systemic infection to occur prior to molecular detection. Total RNA was then extracted from the frozen seedlings using the CTAB-Trizol method (Jordon-Thaden et al., 2015). Retro-transcription was performed using 300 ng/µL of RNA, and ToBRFV was detected by nested RT-PCR using the primers TobN up3 (GGCGYTGCARACIATHGTITAYCA), TobN do4 (GTRTTICCIATRAAIGTIGTIACRTC), and TobN do4G (GCCGATRAAGGTGGTGACRTC), following the protocol of Dovas et al. (2004). Amplified products (~400 bp) were resolved by 1.5% agarose gel electrophoresis and subsequently sequenced by Macrogen^®^ Seoul, Korea. In parallel, 15 subsets of 150 seeds collected from ToBRFV-infected plants were analyzed directly for viral presence, along with seeds from mock-inoculated controls, to assess potential seed-borne transmission.

#### 2.6.3 Infectivity tests of viral particles in seeds

We conducted infectivity evaluations using fifteen subsets, each containing 100 *Nicotiana rustica* seeds. Seeds were surface-disinfected using 3% sodium hypochlorite for three minutes, while parallel tests were conducted on untreated seed subsets for comparison. Following disinfection, seeds from each group were macerated in liquid nitrogen. The resulting homogenate was divided equally into two aliquots: one mixed with 500 µL of phosphate buffer (pH 7.0), and the other with 600 µL of 2% CTAB buffer. The phosphate-buffer-based extracts were used to mechanically inoculate healthy *N. rustica* plants using carborundum as an abrasive. After inoculation, the plants were incubated in a greenhouse under controlled conditions (18–23°C; 12-hour light/dark photoperiod) to allow systemic infection to occur. Plants were monitored daily for symptom development. After a 30-day incubation period, apical leaves were harvested from each plant, and total RNA was extracted using the CTAB-Trizol method. Nested RT-PCR was subsequently performed, as detailed in section 2.6.2, to detect the presence of ToBRFV. This incubation period was essential to allow viral replication and systemic movement before molecular detection. Control groups included two subsets of 100 disinfected seeds, 100 untreated seeds, and 100 seedlings derived from healthy *N. rustica* plants, as illustrated in **Supplementary Figure S4**.

## 3 Results

Tomato plants (*S.lycopersicum* L.) exhibiting classic symptoms of ToBRFV infection—chlorosis, leaf narrowing, and mosaic patterns—were collected from commercial greenhouses in Mexico. RT-PCR confirmed the presence of ToBRFV in the symptomatic samples. Genomic sequencing of the ToBRFV isolate from Mexico (accession number PQ628197) produced 50,136,646 raw reads with a GC content of 50.15% and high-quality scores (Q20: 97.03%, Q30: 92.37%) (**Supplementary Table S1**). After quality trimming, 44,518,584 reads remained, retaining a GC content of 50.35% and achieving improved quality scores (Q20: 98.93%, Q30: 95.61%). Further filtering yielded a final dataset of 99,338 reads, with a GC content of 50.34%. Mapping analysis revealed that 31.41% of the reads aligned to the reference genome NCBI: MW349655.1 Tomato brown rugose fruit virus (6,379 bp), achieving 100% coverage with an average depth of 607.16× (**Supplementary Table S2**). Contig assembly generated a single contig of 6,375 bases with a GC content of 41.54%, demonstrating high sequencing and assembly fidelity (**Supplementary Table S3**; **Supplementary Figure S5**). These results provide a solid foundation for subsequent genomic and computational analyses to elucidate the genetic characteristics and transmission dynamics of this ToBRFV isolate from Mexico designated as accession: BankIt2895689; Mexican PQ628197 in the NCBI database.

The annotated genome of this ToBRFV isolate from Mexico (BankIt2895689 Mexican PQ628197) revealed key protein domains essential for viral replication, transcription, and host interaction (**Table 1**). The viral methyltransferase domain that mapped to the 280–1365 nt in the ToBRFV complete genome, (pfam01660, E-value: 9.92E-08) is critical for RNA capping, enhancing RNA stability and host translation compatibility. The viral helicase domain corresponds to the 2543–3315 nt, (pfam01443, E-value: 4.31E-19) and it is related to supporting RNA unwinding during replication and transcription. Two overlapping RNA-dependent RNA polymerase (RdRp) domains were identified *Virgaviridae_RdRp*, belonging to 3514–4803 nt, (cd23251, E-value: 2.02E-97); and *RdRP_2* corresponding to the 3565–4887 nt of the ToBRV genome, (pfam00978, E-value: 1.04E-96), which is critical for viral RNA synthesis. The viral movement protein (MP) domain in the 4907–5392 nt, (pfam01107, E-value: 8.28E-25) enables intercellular virus transport through plasmodesmata. These findings confirm the presence of highly conserved and functionally significant domains essential for the pathogenicity and life cycle of ToBRFV.

**Table 1:** Annotated functional domains in the genome of the ToBRFV isolate from Mexico.

Name	Accession	Description	Interval (nucleotides)	E-value
Viral Methyltransferase	pfam01660	Viral methyltransferase; This RNA methyltransferase domain is found in a wide range of ssRNA.	280-1365	9.92E-08
Viral_helicase1	pfam01443	Viral (Superfamily 1) RNA helicase; Helicase activity for this family has been demonstrated.	2543-3315	4.31E-19
Virgaviridae_RdRp	cd23251	RNA-dependent RNA polymerase (RdRp) in the family *Virgaviridae* of positive-sense.	3514-4803	2.02E-97
RdRP_2	pfam00978	RNA dependent RNA polymerase; This family may represent an RNA dependent RNA polymerase.	3565-4887	1.04E-96
MP	pfam01107	Viral movement protein (MP); This family includes a variety of movement proteins (MPs).	4907-5392	8.28E-25

Functional domains identified in the ToBRFV genome based on conserved protein families. Predicted protein domains were identified using NCBI blastp webtool that compares against Pfam and Conserved Domain Database (CDD). The table lists domain names, accession IDs, functional descriptions, nucleotide coordinates (interval), and corresponding E-values. These domains include the viral methyltransferase, RNA helicase (Superfamily 1), RNA-dependent RNA polymerase (RdRp), and the movement protein (MP), all characteristic of tobamoviruses.

### 3.1 High genetic similarity and limited divergence of the ToBRFV isolate from Mexico among global variants

**Figure 1:**
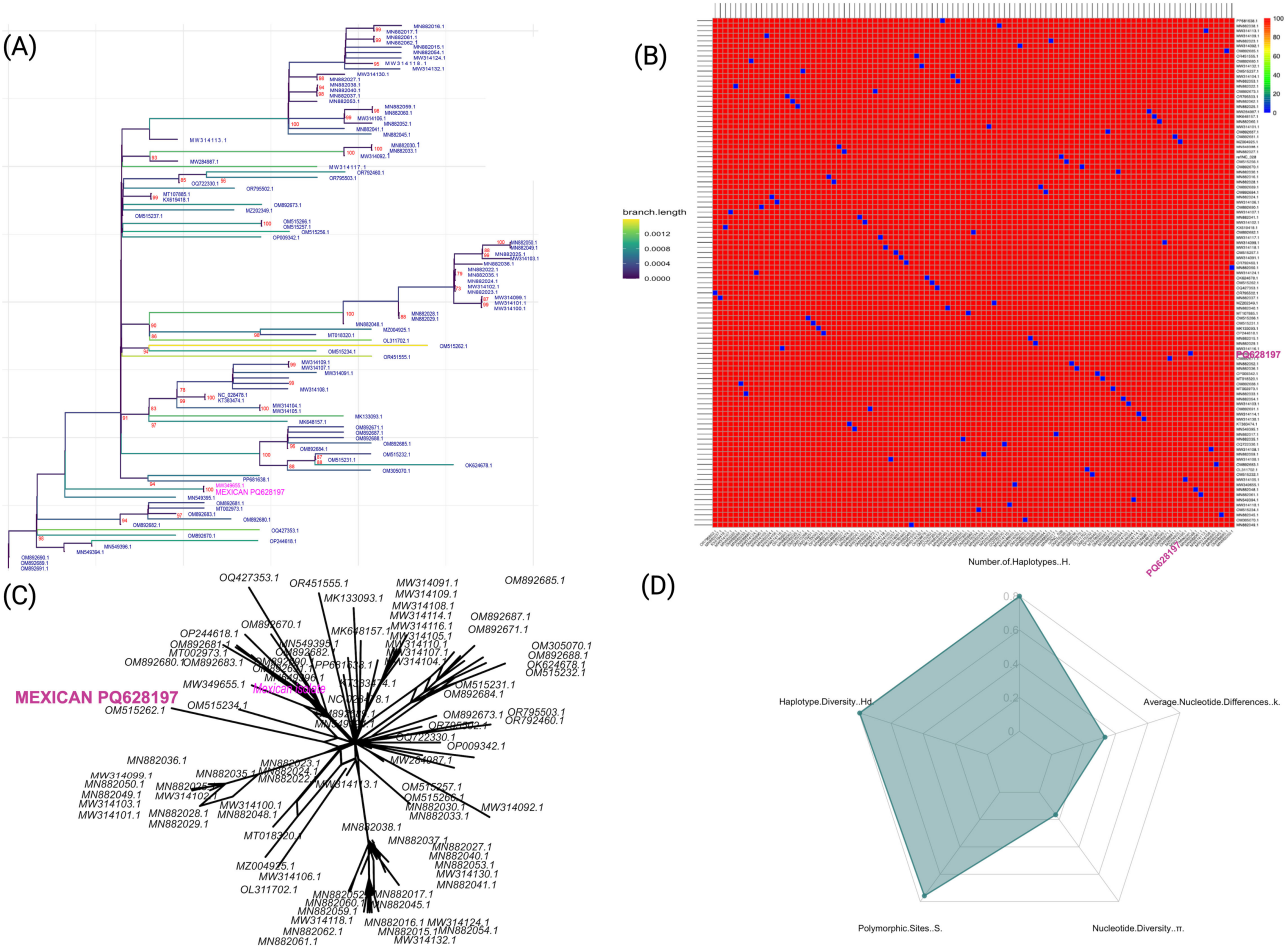
Genetic diversity and phylogenetic analysis of ToBRFV isolates, highlighting the Mexican isolate (NCBI: PQ628197). **(A)** Maximum-likelihood phylogenetic tree with 1000 bootstrap replicates, generated in IQ-TREE2. The Mexican isolated PQ628197 is highlighted in pink, emphasizing its relationship to other isolates. Branch lengths are color-coded by distance to reflect sequence divergence. **(B)** Pairwise sequence identity heatmap for ToBRFV isolates, where colors represent nucleotide identity percentages, with red color indicating higher conservation. **(C)** Neighbor-Net phylogenetic network depicting relationships among ToBRFV isolates, with the Mexican isolate labeled in pink. This network visualizes potential lineage splits and diversification events. **(D)** Radar plot displaying genetic diversity parameters across isolates, including haplotype diversity (Hd), number of haplotypes (H), polymorphic sites (S), nucleotide diversity (π), and average nucleotide differences (k), illustrating the high genetic diversity and core conservation of the virus.

To place the ToBRFV isolate (BankIt2895689 Mexican PQ628197) within a global evolutionary framework, we conducted a comprehensive comparative analysis against 100 publicly available ToBRFV genome sequences. This approach allowed us to understand the isolates genetic relationships, potential transmission features, and evolutionary stability. Our results revealed that the Mexican isolate closely clusters with other genomes, displaying high genetic similarity and suggesting a recent common ancestry with minimal divergence. High bootstrap values across the phylogenetic tree underscore the low variability among these isolates. This high conservation level highlights the stable nature of the ToBRFV genome across diverse geographical regions and hosts (**Figure 1A**). This Mexican isolate (BankIt2895689 Mexican PQ628197) showed close genetic relationships with five other isolates including three collected within Mexico, pointing to recent divergence events and supporting the possibility of local transmission. The phylogenetic tree constructed from the alignment of 100 complete ToBRFV genomes revealed the evolutionary position of the Mexican isolate PQ628197 relative to globally reported sequences. This isolate clustered directly with MW349655.1 from Hidalgo Mexico, sharing a strongly supported node (bootstrap = 100) and exhibiting an extremely low genetic distance (0.00000445), indicating near-identical evolutionary similarity. In addition, the sequences MN549394.1 (Canada), OM892691.1 (USA), OM892690.1 (USA), and OM892683.1 (USA) were positioned within the same subclade, displaying patristic distances ranging from 0.00111749 to 0.00111750 with respect to the Mexican isolate. These five genomes represent the closest phylogenetic relatives to PQ628197, suggesting potential geographic linkage or shared transmission pathways among these isolates.

Collectively, these findings could suggest a pattern of localized transmission within Mexico and the potential for global spread through international seed exchange, emphasizing the role of agricultural practices in the virus’s dissemination. A detailed genetic diversity analysis further contextualizes the Mexican isolate evolutionary position (**Figure 1B**). Pairwise sequence identity calculations show strong nucleotide conservation across the 100 ToBRFV genomes, with the Mexican PQ628197 displaying high sequence similarity to other isolates from Mexico and Israel. This high conservation level, with an average sequence identity of ~99.79%, suggests minimal genetic divergence across them, reinforcing the stability of the ToBRFV genome across diverse locations and hosts. To examine relationships more flexibly, we generated a Neighbor-Net phylogenetic network (**Figure 1C**). The limited branching in this network suggests a pattern of localized transmission within Mexico and supports the hypothesis of international spread through seed trade. This network view aligns with the high sequence identity findings, highlighting a viral population that maintains stability while adapting to new environments with only minor genetic variations. Finally, genetic diversity metrics (**Figure 1D**) provide further insights, showing a pattern of high haplotype diversity (*Hd = 0.994*) with a significant number of unique haplotypes (*H = 80*) among the isolates. This high haplotype diversity indicates substantial genetic variation within the population. Despite this, both nucleotide diversity (*π = 0.0021*) and average nucleotide differences (*k = 0.2079*) remain low, suggesting that sequence differences are generally subtle and the ToBRFV genome remains highly conserved. The observed combination of high haplotype diversity and low nucleotide diversity reflects a population shaped by recent evolutionary and demographic events. This pattern suggests a history of rapid demographic expansion following a population bottleneck, where mutations accumulated in closely related haplotypes. Alternatively, purifying selection may have removed deleterious mutations, constraining nucleotide variability while allowing the proliferation of distinct haplotypes.

In summary, our findings suggest that ToBRFV genomes, including the Mexican isolate, have evolved a balanced strategy of adaptability and stability, with sufficient genetic diversity to respond to environmental pressures while maintaining a highly conserved genome. This evolutionary pattern, likely driven by stabilizing selection, allows the virus to persist and spread effectively across regions.

### 3.2 Geographic and host-specific divergence in the ToBRFV isolate from Mexico

**Figure 2:**
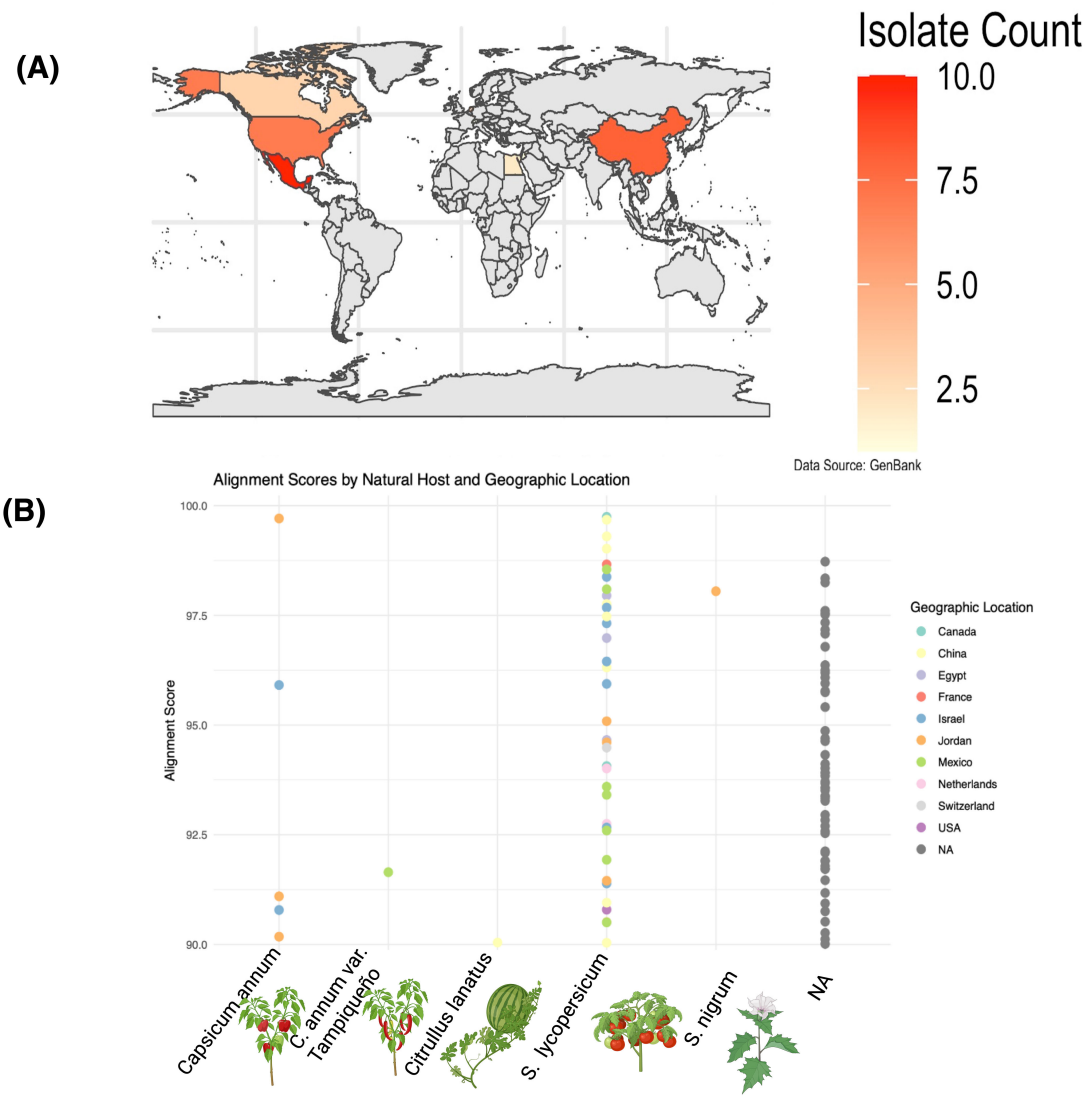
Geographic distribution and alignment scores of ToBRFV (NCBI: PQ628197) isolates across hosts and regions. (**A**) World map indicating the number of ToBRFV isolates per country deposited in GenBank. Countries are shaded according to isolate count, with the highest numbers observed in Mexico, the United States, and China. (**B**) Alignment scores of ToBRFV genomes across different natural hosts, color-coded by geographic location. Each point represents a GenBank-deposited isolate. Isolates derived from Solanum lycopersicum (tomato) show higher genetic similarity with the Mexican isolate used as reference, while those from Citrullus lanatus (watermelon) and Capsicum annuum (pepper) display lower alignment scores, suggesting host-specific divergence. Geographically, isolates from Mexico and the USA exhibit the highest similarity, indicating strong regional conservation. Data source: NCBI GenBanK.

The geographical clustering of ToBRFV isolates highlights regional patterns that suggest localized adaptation and potential evolutionary divergence. Clades formed by isolates from distinct regions, such as Israel and Europe, suggest the presence of regional signatures potentially influenced by geographic or host-related factors. Within this context, this Mexican isolate (NCBI accession: BankIt2895689 Mexican PQ628197) exhibits a unique genetic position, balancing distinctiveness and similarities with geographically distant isolates. The geographical distribution of genetically aligned isolates (**Figure 2A**) reveals affinities with this Mexican strain in regions such as Mexico, Israel, and China, while lower alignment frequencies in areas like Jordan, and the United Kingdom suggest varied evolutionary pressures and degrees of relatedness. Host-specific analyses provided additional insights into ToBRFV’s genetic variability and adaptive mechanisms (**Figure 2B**). Isolates from *S. lycopersicum* (tomato), the host of the Mexican reference genome (NCBI accession: BankIt2895689 Mexican PQ628197), displayed high alignment scores, reflecting stable adaptation and a conserved genetic profile within this primary host. This suggests a recent shared ancestry between the Mexican isolate and other tomato-derived isolates. In contrast, isolates from *C. annuum* (pepper), and *C. annuum* “*tampiqueño*” variety, showed moderate alignment scores, indicative of genetic divergence driven by host-associated sequence variation potentially shaped by selective pressures. In our analysis, greater variability was observed in *C. lanatus* (watermelon) isolates, suggesting evolutionary adaptations that enhance ToBRFV fitness in non-tomato hosts. These patterns of alignment scores by host and geographic location (show **Figure 2B**) that ToBRFV may follow distinct evolutionary paths in different host environments, potentially influenced by host defense mechanisms. This highlights the complexity of its interaction landscape and points to the potential relevance of host-specific considerations in management strategies.

### 3.3 Genomic divergence in ToBRFV replicase regions: exploring potential host-associated variations

**Figure 3:**
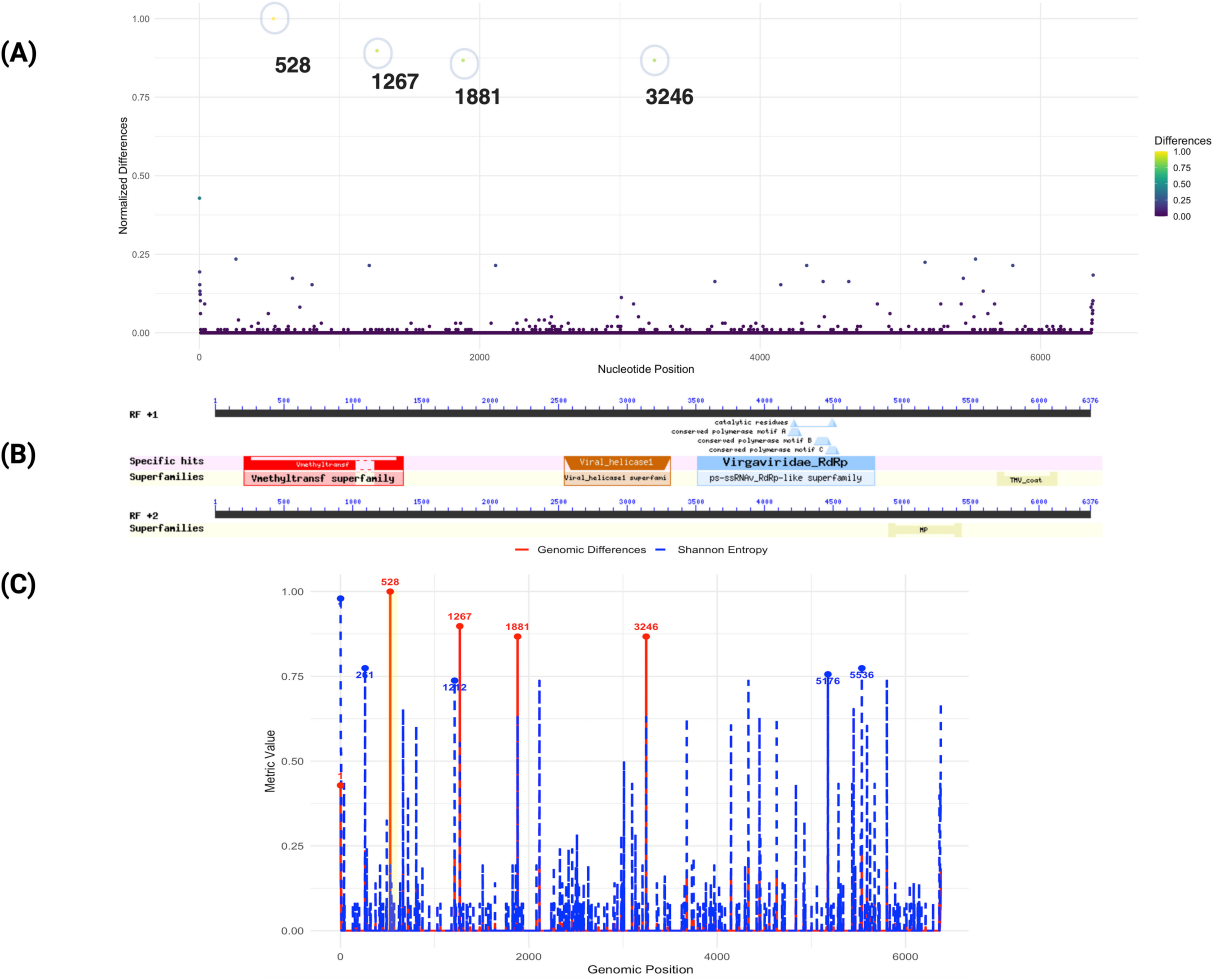
Genomic differences of ToBRFV isolate from Mexico compared to 100 public genomes. **(A)** This plot displays normalized nucleotide differences across the ToBRFV isolate in comparison to 100 other isolated genomes. Each point represents a nucleotide position, with the y-axis indicating normalized difference values and the x-axis showing nucleotide positions. Colors reflect the degree of variation, from low (purple) to high (yellow). Notable peaks at positions 528, 1267, and 1881 reveal regions of high genetic divergence, which correspond to genomic features associated with viral replication. **(B)** illustrates the specific functional domains identified within the ToBRFV genome. Superfamilies and conserved motifs are marked, demonstrating key structural and functional elements within the genome; **(C)** Comparison of genomic differences and Shannon entropy across the *ToBRFV* genome. The red solid line represents genomic differences, while the blue dashed line shows Shannon entropy. Key positions with the highest values for each metric are highlighted and annotated. A highlighted yellow region indicates an area of specific interest.

To explore potential genomic differences in the ToBRFV PQ628197, we conducted a genome comparative analysis focusing on specific nucleotide positions that exhibited significant variation in contrast to the 100 ToBRFV genomes. This approach focused on nucleotide positions showing higher normalized divergence values (**Figure 3**). We identified five positions with elevated variation, which may reflect regions prone to mutation or possibly influenced by evolutionary processes. Notably, the most variable position was observed at nucleotide 528, where a normalized difference of 1.000 suggests complete divergence at this site among the isolates (**Figure 3A**). Similarly, positions 1267 and 1881 displayed high levels of variability, with normalized differences of 0.897 and 0.867, respectively. The region surrounding nucleotide positions 528 and 1267 corresponds to the genomic span of 280–1365, which encodes a viral methyltransferase domain (*pfam01660*). This domain is involved in RNA cap methylation, enhancing RNA stability and ensuring compatibility with the host’s translation machinery. Variability in these positions may impact the efficiency of these processes, possibly influence viral fitness and contributing to minor adaptive differences. Variations within this protein-coding region may influence the virus’s fitness by affecting replication efficiency or interactions with host defenses, particularly if ToBRFV is adapting to new hosts or undergoing selection in greenhouse settings with intensive agricultural practices. Such adaptations could contribute to the virus’s persistence and ability to infect a diverse range of host species.

The region surrounding nucleotide position 1881 is not directly associated with any of the annotated functional domains in the current analysis. However, its high variability suggests it may correspond to an uncharacterized functional element or a site under evolutionary pressure. Nucleotide position 3246, with a normalized difference of 0.867, is located within the viral helicase domain (*pfam01443*, spanning 2543–3315, E-value: 4.31E-19), part of ORF1 (**Figure 3B**). This domain is essential for RNA unwinding during replication and transcription, highlighting its critical role in the viral life cycle. The observed variation at this position may reflect selective pressures acting on the helicase function, potentially enhancing the virus’s adaptability and infectivity across diverse hosts and environments. These findings indicate that the replicase coding regions, particularly ORF1, represent hotspots for genomic variation in the ToBRFV isolate from Mexico. Variability within key domains, such as the methyltransferase and helicase domains, may play a pivotal role in the virus’s adaptation strategies, enabling it to optimize replication and transmission in diverse ecological contexts (**Figure 3C**). Shannon entropy analysis further revealed distinct variability patterns. A moderate positive Pearson correlation (0.66) and an almost perfect Spearman correlation (0.9999) between genomic differences and entropy suggest that higher sequence divergence aligns with greater sequence variability, **Supplementary Figure S6**. Notably, position 1, with the highest Shannon entropy (0.979) and a significant genomic difference (0.429), emerges as a critical hotspot of genomic diversity with potential functional relevance. While position 528 exhibits the highest genomic difference (1.000) but low entropy (0.193), indicating unique divergence specific to the Mexican isolate, positions with high entropy but low differences (e.g., 261, 5536) may represent conserved regions with broader alignment variability. Adaptive hotspots such as 1267, 1881, and 3246, characterized by both high differences (~0.87) and moderate entropy (~0.63), likely reflect regions under balanced evolutionary forces. These insights underscore the functional and evolutionary importance of replicase coding regions, particularly ORF1, in shaping the virus’s ecological adaptation and host interactions.

#### 3.3.1 Single nucleotide variation patterns in ToBRFV replicase regions across different hosts

**Figure 4:**
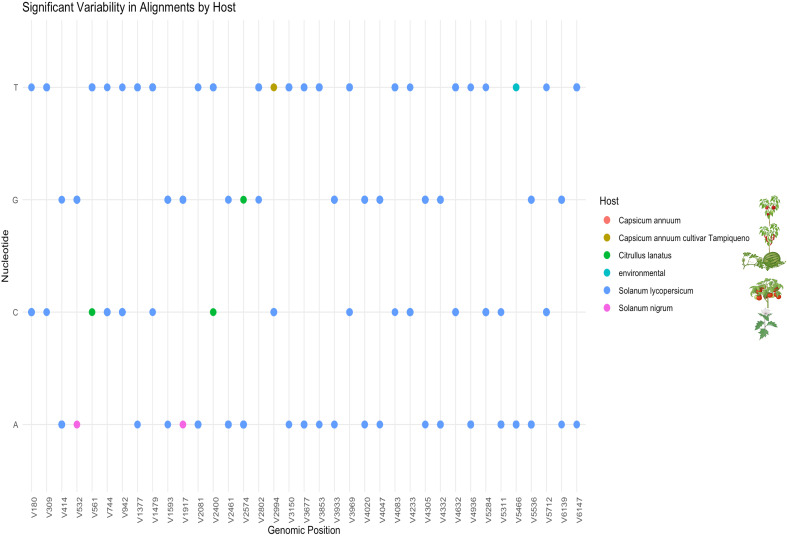
Significant nucleotide variability in ToBRFV isolates by host. This figure shows specific nucleotide positions in the ToBRFV genome where variability was observed across different hosts. Dot plot showing the distribution of host-associated SNVs identified across the ToBRFV genome. The x-axis denotes genomic positions where SNVs were detected (labeled as V# for each variant), and the y-axis represents the nucleotide observed (A, C, G, T). Each dot corresponds to a nucleotide at a given SNV position, color-coded according to the host species: Capsicum annuum, C. annuum cv. Tampiqueño, Citrullus lanatus, Solanum lycopersicum, Solanum nigrum, and environmental samples. The majority of variants are observed in isolates from S. lycopersicum (blue), while host-specific SNVs in other species may suggest unique polymorphisms or early adaptation events.

The detailed nucleotide variability analysis provides insights into potential host-associated patterns of evolution and adaptability in ToBRFV. Isolates from *S. lycopersicum* exhibited highly conserved nucleotide profiles, particularly at positions (V=variant) V180, V414, V744, and V1377 within the replicase regions (**Figure 4**). This conservation underscores the virus’s genetic stability in its primary host and may reflect a host-associated evolutionary trajectory that has contributed to optimizing ToBRFV’s replication and transmission efficiency in tomato. Such stability indicates that the virus has reached a near-equilibrium state in this host, reflecting selective pressures that maintain essential genetic features critical for fitness. In contrast, isolates from alternative hosts, including*, C. annuum* var. *“tampiqueño”*, *S. nigrum* and *C. lanatus*, displayed unique single nucleotide variants (SNVs) which may reflect host-associated sequence variation potentially shaped by selective pressures. For instance, substitution at position V2994 in *C. annuum* var. *“tampiqueño”* showing variability, likely due to localized ecological and molecular pressures. In *C. lanatus* (watermelon), the identification of three notable SNVs at positions V561, V2400, and V2574 demonstrates a higher degree of genetic divergence. The substitution at position V561, located within the viral methyltransferase domain of the 126 kDa replicase protein, is of particular interest due to its potential impact on RNA cap methylation, a process critical for RNA stability and translation. The remaining substitutions fall outside annotated functional host-specific barriers in *C. lanatus*. These substitutions may reflect evolutionary attempts by ToBRFV to establish infection in a less compatible host, highlighting the challenges the virus faces in adapting to non-tomato hosts. The greater variability observed in watermelon isolates underscores the incomplete adaptation process, which could involve modifications to critical genomic regions such as the replicase or other viral proteins. Interestingly, *S. nigrum* isolates showed only two significant SNVs, both featuring adenine (A) at positions V532 and V1917 within the coding region of the 126 kDa replicase protein. These positions overlap with the viral methyltransferase domain, suggesting a potential impact on replication efficiency. The limited variability in *S. nigrum* compared to other alternative hosts might reflect its role as a less favorable host, where selective pressures are weaker or less sustained due to limited viral replication or transmission success. In *C. annuum*, the thymine substitution observed at position V2994 within the helicase protein domain is particularly notable. These findings highlight the complex interplay between the virus and host-specific factors, driving genetic adaptations in non-primary hosts.

Overall, the presence of host-associated SNVs within the replicase regions suggests a degree of genetic plasticity in ToBRFV, although the complete identity observed between isolates from different hosts also points to a high level of genome conservation and broad host compatibility. Variability within key functional domains, such as the methyltransferase, and helicase regions, may provide with the flexibility to modulate replication dynamics and host interactions. While *S. lycopersicum* isolates exhibit most of the identified SNVs, these variants are widely distributed across positions and likely reflect intra-host variation within a well-established viral population (**Figure 4**). In contrast, isolates from alternative hosts such as *C. lanatus, C. annuum var “tampiqueño’*, and *S. nigrum* display a limited number of unique SNVs at specific genomic positions, this could reflects signals of host-associated divergence potentially influenced by distinct selective environments or transmission dynamics.

**Figure 5:**
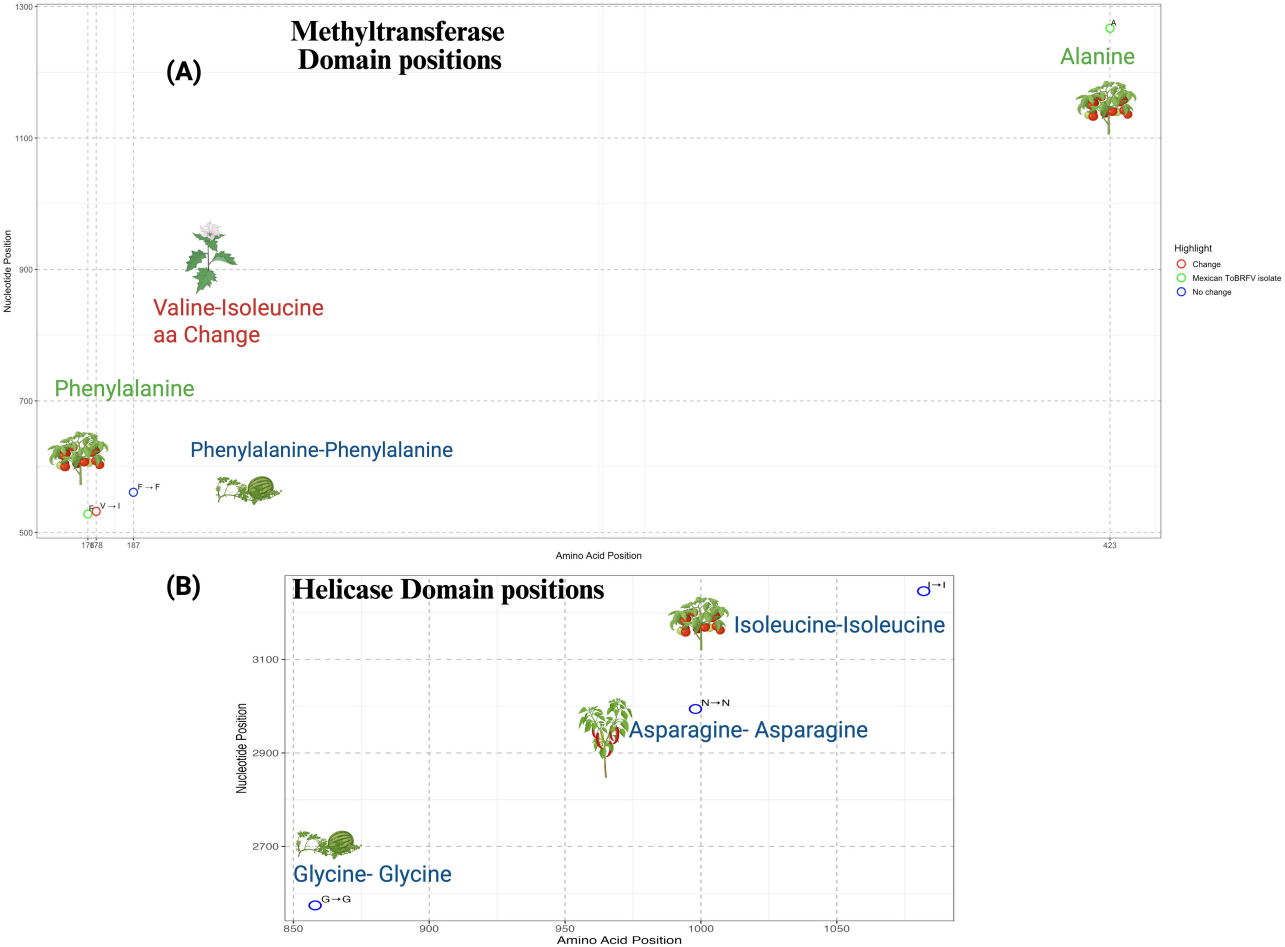
Amino acid variation within functional domains of the ToBRFV replicase. **(A)** Distribution of amino acid positions and corresponding nucleotide coordinates within the methyltransferase domain of the ToBRFV replicase. Conserved residues are shown in blue, amino acid-invariant positions at highly variable nucleotide sites specific to the Mexican isolate are shown in green, and a non-synonymous substitution (Valine to Isoleucine) unique to the Solanum nigrum host is highlighted in red. **(B)** Equivalent plot for the helicase domain, where all analyzed isolates—including the Mexican isolate—exhibit conserved amino acids. No amino acid substitutions were detected in this domain. Both panels plot amino acid positions along the x-axis and nucleotide coordinates along the y-axis. Colored circles denote positions with no variation (blue), specific changes in the Mexican isolate (green), or non-synonymous substitutions (red). The corresponding host for each mutation is visually represented using an icon.

All the genomic differences identified within coding regions fall within annotated replicase domains. To assess the potential functional consequences of these mutations, we conducted an amino acid substitution analysis to evaluate how the observed changes might influence the structural or functional integrity of the affected domains (**Figure 5**). The detailed amino acid substitution analysis, particularly within the methyltransferase domain of the replicase region, offers valuable insights into potential molecular adaptations of ToBRFV related to host interactions (**Figure 5A**). Amino acid positions 176 corresponding to the V528 nucleotide position **Figure 3**, as well as 423 from V1267, identified as the most divergent to the Mexican isolate, emphasize its genetic distinctiveness (**Figure 5A**). These positions, which correspond to nucleotide changes within the methyltransferase domain, may be shaped by local factors, although further evidence is needed to determine their adaptive significance. Position 176, in particular, resides within a region critical for RNA cap methylation, a key process required to stabilize viral RNA and ensure efficient translation. Although the functional implications remain to be fully understood, variation at this site may play a role in maintaining ToBRFV’s replication efficiency in *S. lycopersicum.* Aminoacidic position 178 (V532), characterized by a valine-to-isoleucine substitution, may represent a case of host-associated variation, particularly in *S. nigrum*, potentially reflecting early signals of adaptation. This mutation likely enhances viral infectivity by optimizing interactions with host-specific cellular machinery, supporting the hypothesis that ToBRFV undergoes selective pressures to overcome barriers in alternative hosts. Conversely, position 187 aa (V561) remains conserved as phenylalanine in *C. lanatus*, suggesting functional constraint or stability at this site. The lack of variability in *C. lanatus* indicates weak selective pressures, potentially reflecting limited evolutionary interaction or incomplete adaptation to cucurbit hosts.

To further explore the functional implications of the Valine-178-Isoleucine substitution, we modeled this mutation using DynaMut2 and Swiss-Model. The substitution, which maps to position 849 in the predicted structure, resulted in a predicted ΔΔG of –0.84 kcal/mol, indicating a destabilizing effect on protein stability. This was supported by I-Mutant2.0, which also predicted a decrease in stability (RI 5), and PROVEAN, which classified the substitution as neutral (score –0.396) (**Table 2**). The structural model (**Supplementary Figure S6**) reveals altered local flexibility surrounding the mutation site. These computational results suggest that the Valine-178-Isoleucine substitution may influence the structural flexibility of the replicase protein, which could, in turn, affect viral replication or interactions with the host in *S. nigrum.* The analysis further highlights conserved positions outside annotated functional domains, such as position 3100, where no significant amino acid changes were detected. We also explored the presence of possible aminoacidic changes within the helicase region, **Figure 5B**. We focused on the SNV at position V2574, detected in *C. lanatus*, the SNV at V2994 in *C. annuum “tampiqueño”*, and included the V3246 region from *S. lycopersicum.* Although nucleotide variability was observed, our analysis did not reveal any non-synonymous mutations within these regions. This suggests that they may play important structural or regulatory roles, contributing to the stability of the replicase protein. Their high conservation highlights their relevance in maintaining the functional robustness of ToBRFV across diverse hosts. Despite the lack of sequence divergence, these regions likely form part of the core replicative machinery essential for viral propagation.

**Table 2:** *In silico* predictions of the structural and functional impact of the V178I mutation in the methyltransferase domain of ToBRFV replicase.

Tool	Position in structure	Prediction	Score/ΔΔG	Interpretation
I-Mutant2.0	178 sequence	Decrease in stability	RI = 5	Likely destabilizing
PROVEAN	178 sequence	Neutral	Score = –0.396	Tolerated mutation
DynaMut2	178 = 849 3D structure	Destabilizing effect on conformation	ΔΔG = –0.84 kcal/mol	Reduced thermodynamic stability
SwissModel	178 (3D structure)	Predicted structural flexibility change	RMSD = 0.832 Å	Suggests altered local conformation

*In silico* predictions assessing the structural and functional impact of the Valine-178-Isoleucine mutation located in the methyltransferase domain of the ToBRFV replicase protein. The position 178 refers to the amino acid sequence position, while 849 corresponds to its mapping in the SwissModel 3D structural context. I-Mutant2.0 predicts a decrease in stability (reliability index = 5), PROVEAN classifies the substitution as functionally neutral (tolerated), and DynaMut2 indicates a destabilizing conformational effect with a ΔΔG of –0.84 kcal/mol. Swiss-Model was used to generate a high-quality 3D model of the replicase protein for structural mapping and visualization.

The interplay between conserved and variable sites within the replicase region highlights ToBRFV’s evolutionary strategy to preserve essential viral functions while allowing adaptive flexibility in alternative hosts. Conserved residues within critical functional domains, such as the methyltransferase, ensure replication efficiency and stability in its primary host, *S. lycopersicum*. In contrast, variable residues identified in *S. nigrum* and *C. lanatus* demonstrate the virus’s capacity for adaptive divergence in response to host-specific selective pressures. The close genetic similarity among Mexican *S. lycopersicum* isolates reflects the evolutionary stability of ToBRFV in tomato hosts, while the variability in non-tomato hosts points to adaptive divergence.

### 3.4 Evidence of expanded host susceptibility of ToBRFV in native and commercial solanaceae

**Figure 6:**
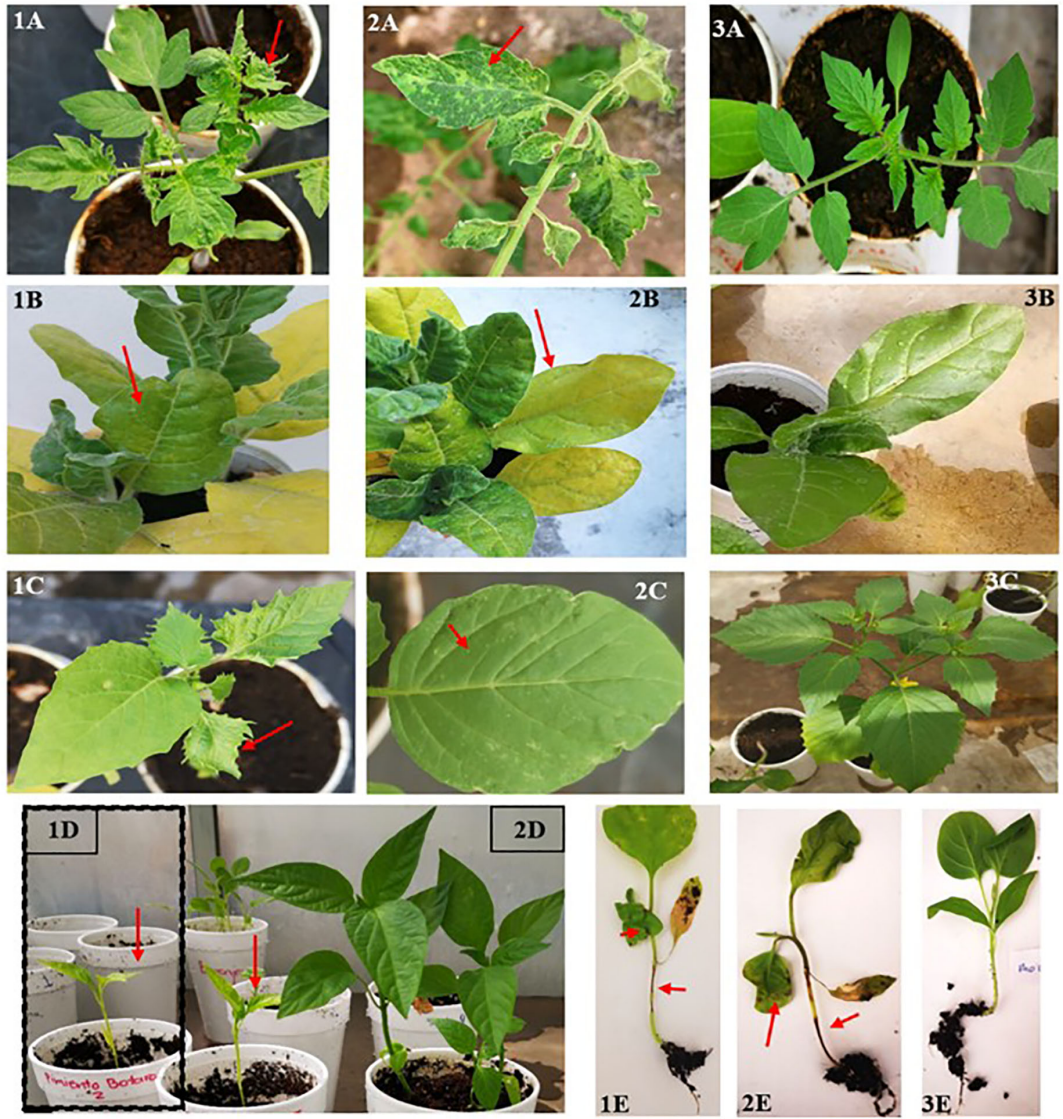
Symptoms in various hosts following mechanical inoculation with ToBRFV. Representative symptoms observed in tomato **(1–2A)**, tobacco **(1–2B)**, tomatillo **(1–2C)**, pepper **(1D)**, and eggplant **(1–2E)** after mechanical transmission of ToBRFV. Infected plants exhibited leaf narrowing **(1A, 1C)**, mosaic patterns **(2A)**, stem and leaf necrosis **(1E, 2E)**, chlorosis **(1B, 2B)**, chlorotic lesions **(2C)**, and stunting **(1D)**. Panels **3A, 3B, 3C, 2D**, and **3E** correspond to mock-inoculated control plants, showing no visible symptoms.

Following the *in silico* analysis of host specificity using available isolates from the GenBank DB, experimental bioassays were performed to assess the susceptibility of various potential hosts to the ToBRFV PQ628197 isolate from Mexico. The tested species included commercially cultivated plants—such as watermelon (*C. lanatus*), cantaloupe (*C. melo*), squash (*C. pepo*), cucumber (*C. sativus*), pea (*Pisum sativum*), tomato (*S. lycopersicum*), and pepper (*C. annuum*)—alongside less commonly cultivated or wild species, such as tomatillo (*P. ixocarpa*), tobacco (*N. rustica*), and eggplant (*S. melongena*).The bioassays revealed strong host specificity for the ToBRFV Mexican PQ628197, with successful replication observed exclusively in solanaceous hosts. Remarkably, the virus failed to replicate in any cucurbit hosts despite multiple inoculation attempts. This lack of replication suggests that this isolate lacks specific genetic adaptations required to efficiently interact with the cellular machinery of cucurbit hosts. As summarized in **Table 3**, our findings confirmed tomato and pepper as susceptible hosts for the ToBRFV isolate from Mexico. In contrast, no symptoms or detectable viral presence were observed in any tested Cucurbitaceae species, including watermelon, cantaloupe, squash, and cucumber. Moreover, experimental testing revealed new susceptibility insights for *N. rustica* (tobacco), *P. ixocarpa* (tomatillo), and *S. melongena* (eggplant). Interestingly, tomatillo and eggplant displayed partial infection, suggesting a degree of susceptibility not previously recorded. This variability in infection response highlights the nuanced host-pathogen interactions within the Solanaceae family, with some individuals within species demonstrating possible resistance or lower susceptibility to ToBRFV. The symptomatic responses observed in infected plants, detailed in **Figure 6**, reflect these differences. Tomato and tobacco displayed classic ToBRFV symptoms, such as leaf narrowing and mosaic patterns, while tomatillo showed narrowing and chlorotic lesions, pepper exhibited stunting, and eggplant showed necrosis on both stem and leaves. In contrast, mock-inoculated plants (**Figures 6A–E**) showed no symptoms, underscoring the specificity of ToBRFV-induced symptoms across susceptible hosts. These findings highlight the importance of experimental validation to better understand ToBRFV’s host range, particularly in endemic and agriculturally significant species. The variability in susceptibility among species underscores the complexity of host-virus interactions and the need for further research into the genetic and environmental factors shaping host susceptibility and resistance.

**Table 3:** Analysis by nested RT-PCR and ELISA of ToBRFV inoculated plants.

Inoculated specie	Nested RT-PCR	ELISA
Watermelon (*Citrullus lanatus*)	0*, 0°, 0”	0, 0, 0
Cantaloupe (*Cucumis melo*)	0, 0, 0	0, 0, 0
Squash (*Cucurbita pepo*)	0, 0, 0	0, 0, 0
Cucumber (*Cucumis sativus*)	0, 0, 0	0, 0, 0
Pea (*Pisum sativum*)	0, 0, 0	0, 0, 0
Tomato (*Solanum lycopersicum*)	5, 5, 5	5, 5, 5
Tomatillo (*Physalis ixocarpa*)	3, 4, 3	3, 4, 3
Tobacco (*Nicotiana rustica*)	5, 5, 5	5, 5, 5
Pepper (*Capsicum annuum*)	2, 4, 5	2, 4, 5
Eggplant (*Solanum melongena*)	0, 1, 5	0, 1, 5

Detection of ToBRFV in inoculated plant species by nested RT-PCR and ELISA.Results of ToBRFV detection in mechanically inoculated plants using nested RT-PCR and ELISA. Each value represents the number of positive detections out of five inoculated plants, across three independent biological replicates. A value of 0 indicates no detection. Tomato, tomatillo, tobacco, and pepper showed consistent or partial infection, whereas other species, including watermelon, cantaloupe, squash, cucumber, and pea, showed no evidence of infection in either assay.

### 3.5 Seed-borne transmission dynamics

**Figure 7:**
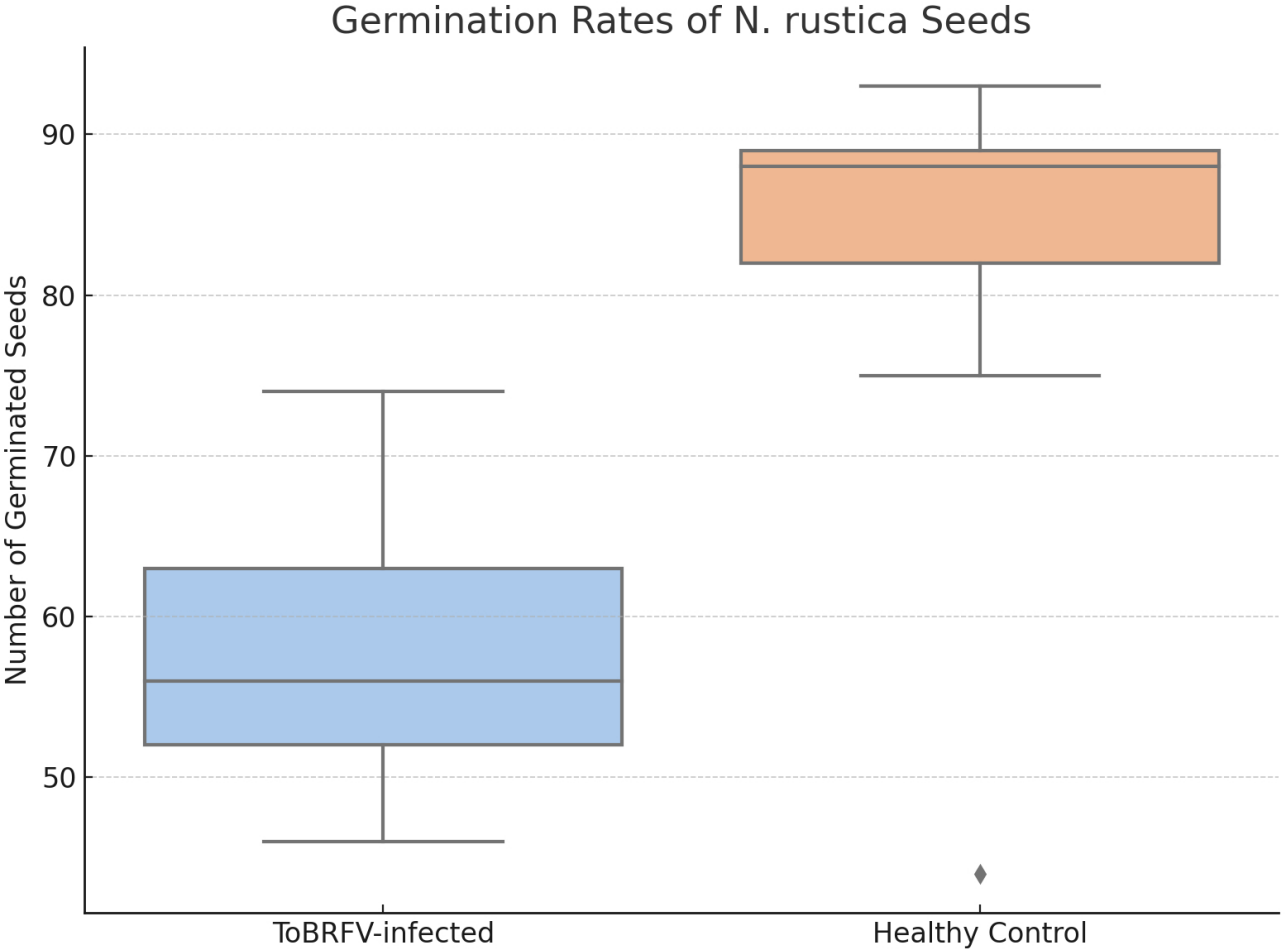
Germination rates of Nicotiana rustica seeds from ToBRFV-infected and healthy plants. Boxplot illustrating the number of germinated *N. rustica* seeds per replicate in two experimental groups: seeds collected from ToBRFV-infected plants and from healthy controls. Each box represents the interquartile range (IQR) with the median indicated by the horizontal line inside the box; whiskers extend to the minimum and maximum values within 1.5× IQR. Black dots represent individual data points (replicates). Statistical comparison using Welch’s t-test showed a significant difference between groups (t(13.36) = –4.36, p = 0.0008), confirming that viral infection significantly impairs seed germination capacity.

Further investigations into seed-borne transmission in tobacco, a known ToBRFV-susceptible species, provided valuable insights due to its suitability for controlled bioassays. To examine the effect of ToBRFV infection on seed viability, we analyzed germination rates in seeds from ToBRFV-infected and control *N. rustica* plants. The results demonstrated a significant reduction in germination rate for seeds from infected plants, with a mean germination rate of 57.7% compared to 82% for control seeds (**Table 4**). This decrease suggests that ToBRFV infection adversely affects the reproductive success of *N. rustica* by reducing seed viability. This finding aligns with earlier analyses suggesting complex host-pathogen interactions were viral infections impact plant reproduction beyond visible symptoms (Hemmati and McLean, 1977). A comparative analysis of seed germination revealed a significant reduction in germination rates in seeds derived from *N. rustica* ToBRFV-infected plants compared to healthy controls. **Figure 7** displays the distribution of germinated seeds per replicate in both groups. Seeds from infected plants exhibited a markedly lower median germination, with reduced interquartile range, indicating both lower and more variable performance. Statistical analysis using Welch’s t-test confirmed a significant difference between the two groups t (13.36) = –4.36, p = 0.0008, demonstrating that ToBRFV infection negatively impacts the germination capacity of *N. rustica* seed.

**Table 4:** Comparison of germination rates: *N. rustica* seeds from healthy versus ToBRFV-infected plants.

	Number of germinated seeds	
Replicate	ToBRFV-infected	Healthy-control
1	58	92
2	56	87
3	64	88
4	56	75
5	74	88
6	52	82
7	46	44
8	63	89
9	51	93

Number of germinated *Nicotiana rustica* seeds per replicate from ToBRFV-infected and healthy control plants. Each replicate consisted of 100 seeds. A significant reduction in germination was observed in seeds from ToBRFV-infected plants.

#### 3.5.1 ToBRFV infection reduces seed viability and enables potential seed-borne transmission in wild species, posing biosecurity risks

**Figure 8:**
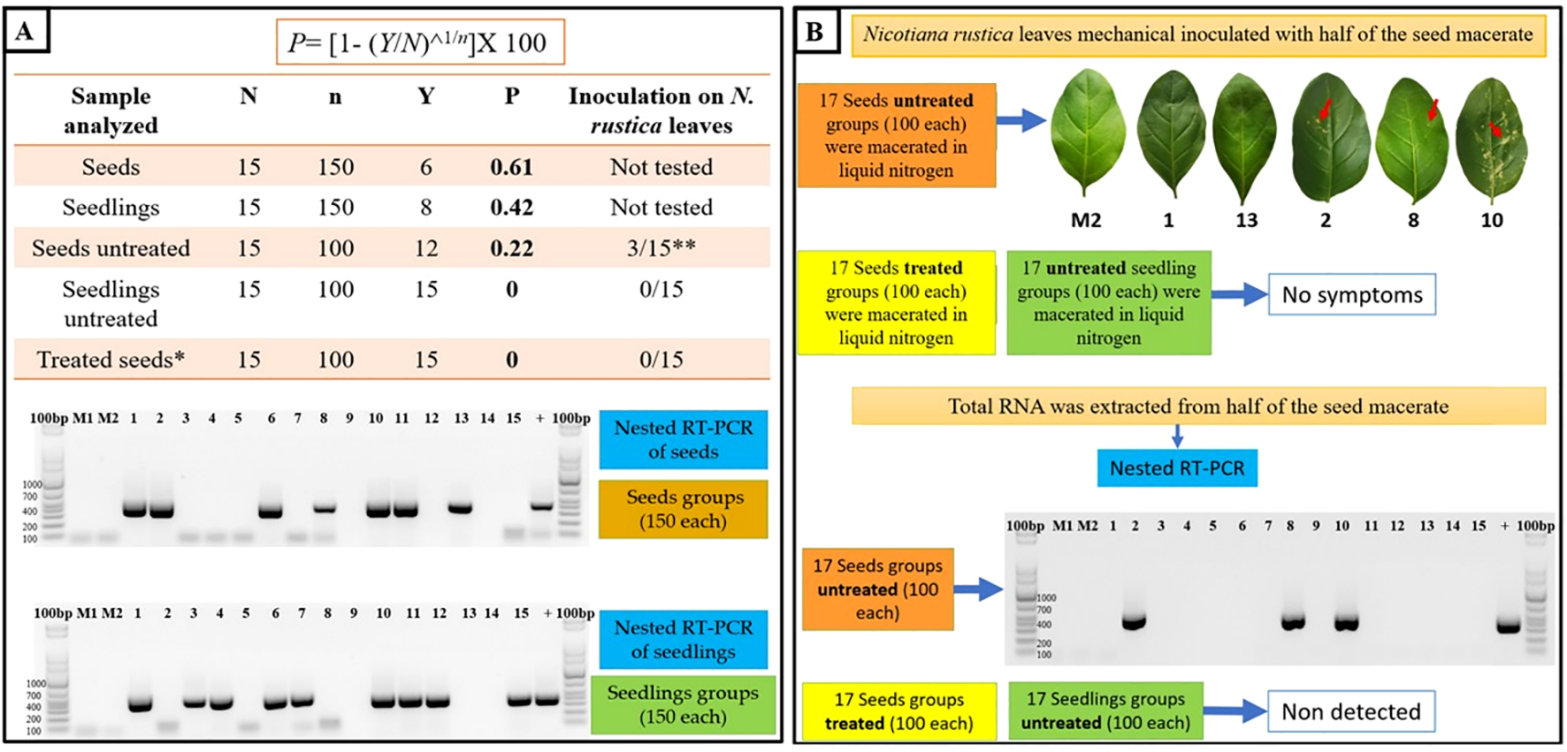
Seed-borne transmission and infectivity analysis of ToBRFV in *N. rustica*. **(A)** Seed transmission dynamics were evaluated through germination and nested RT-PCR analyses on *N. rustica* seeds and seedlings from ToBRFV-infected plants. Seed subsets with and without 3% sodium hypochlorite treatment were tested, and infection rates (P) were calculated using the formula: P=[1−(Y/N)1/n], where N is the total number of subgroups, n is the number of seeds/seedlings per subgroup, and Y represents RT-PCR-negative subgroups. The electrophoretic gel image displays RT-PCR products (400 bp) for ToBRFV detection in infected and uninfected (control) samples, with M1 and M2 as healthy plant controls.**(B)** Viral infectivity in *N. rustica* was assessed by inoculating leaves with seed macerates from infected plants. Chlorotic lesions (arrow) indicate symptomatic infection, confirmed by RT-PCR (400 bp band). Treated seeds (sodium hypochlorite) showed no RT-PCR detection or symptoms, underscoring the role of surface disinfection in reducing infectivity.

To quantify the prevalence of ToBRFV within *N. rustica* seeds and seedlings, nested RT-PCR was conducted on varying subsample sizes, offering insights into virus detection variability. The infection rate in 150-seed subsamples was 0.61%, while 100-seed subsamples showed a lower infection rate of 0.22% (**Figure 8A**). Furthermore, ToBRFV presence was detected only in seedlings from the 150-seed subsamples, while no virus was found in seeds treated with a 3% sodium hypochlorite solution. These findings underscore the role of sample size and disinfection treatment in virus detection, highlighting sodium hypochlorite’s efficacy in removing surface-bound virions. However, the persistence of ToBRFV within internal seed tissues suggests that surface disinfection alone may be insufficient, emphasizing the need for rigorous testing protocols in seed transmission studies.

#### 3.5.2 Assessing seed-borne viral infectivity

Bioassays on *N. rustica* seeds revealed that only three groups of inoculated leaves exhibited symptoms, confirmed as ToBRFV-positive via nested RT-PCR (**Figure 8B**). No symptoms or viral presence were detected in seedlings from seeds treated with sodium hypochlorite, highlighting that untreated seeds may harbor viable ToBRFV particles capable of initiating infection under favorable conditions. The reduction in germination rates observed in seeds from ToBRFV-infected plants suggests that the virus may affect seed viability. While the exact mechanisms are not yet clear, ToBRFV could interfere with seed development through host-virus interactions during infection. These results point to a possible impact on crop establishment and highlight the need for further studies to better understand how ToBRFV influences seed physiology and plant propagation.

## 4 Discussion

This study delves into the molecular basis and implications of ToBRFV’s adaptability, highlighting the Mexican isolate (NCBI BankIt2895689 PQ628197) remarkable genetic stability, environmental resilience, and broad host range across commercial crops and endemic species. Its ability to infect diverse Solanaceae species underscores its potential for persistence and spread in agriculturally and ecologically diverse regions like Mexico. This adaptability raises concerns about ToBRFV’s impact on agriculture and its establishment in natural environments, where interactions with a wider host array could drive new infection cycles.

### 4.1 Geographic distribution of ToBRFV: insights into potential transmission pathways

Our results suggest that the ToBRFV isolate from Mexico shares high genomic similarity with strains reported in USA, Canada, China and Israel, indicating possible cross-regional connections. Additionally, independent reports of a second introduction into Mexico, involving a strain related to isolates from the Netherlands and the Middle East (Vargas-Mejía et al., 2023), are consistent with the possibility of multiple entries, potentially associated with international trade in seeds or plant materials. While our data do not directly demonstrate transmission routes, the observed phylogenetic proximity raises questions about long-distance dispersal mechanisms, which may include global agricultural exchange. The presence of genetically distinct ToBRFV strains within Mexico may reflect complex introduction histories or regional diversification. Although recombination or adaptive evolution cannot be confirmed without additional molecular evidence, such genomic variability warrants closer surveillance. These findings reinforce the importance of enhanced monitoring and international cooperation. In particular, the implementation of robust quarantine protocols and seed certification practices may contribute to limiting ToBRFV’s spread, especially in regions with intensive crop production and trade activity.

### 4.2 Genomic stability and host-associated variability of the ToBRFV isolate from Mexico in replicase domains

The genome of the ToBRFV isolate from Mexico demonstrates a strategic balance between genetic stability and adaptive flexibility, enabling infection of diverse hosts with minimal genetic drift. Between 2019 and 2021, field surveys across tomato-producing states in Mexico (Colima, Hidalgo, Michoacán, and Querétaro) confirmed ToBRFV infection in symptomatic plants via RT-PCR. Phylogenetic analysis of RdRp sequences revealed very low nucleotide diversity and no phylogeographic structure among isolates from these regions, indicating a genetically homogeneous ToBRFV population in central-western Mexico (**Supplementary Figure S1**). The Colima isolate sequenced in this study is nearly identical to a previously reported complete genome from Hidalgo, further supporting its representativeness within the predominant Mexican ToBRFV lineage. Moreover, symptom severity correlated more strongly with tomato cultivar than with isolate origin, as cherry tomatoes exhibited more severe symptoms, while *“bola”* and *“saladette”* cultivars displayed milder patterns. These findings are consistent with international reports of limited genetic divergence among ToBRFV populations and reinforce the conclusion that the Colima isolate provides a robust reference for ongoing molecular, epidemiological, and biosecurity studies both within Mexico and in a global context. Comparative analysis of our ToBRFV isolate and 100 global publicly available genomes revealed unique SNVs in the methyltransferase and helicase domains, suggesting host-specific selective pressures. These domains, critical for replication, transcription, and immune suppression, highlight their dual role in preserving genetic integrity and supporting viral persistence across agricultural and natural ecosystems. Our study corroborates recent findings that reveal ToBRFV’s low genetic variability and strong negative selection pressures, a pattern further supported by an analysis of an Iranian isolate (Esmaeilzadeh et al., 2023) which grouped ToBRFV into three clades with minimal genetic variation. This consistency across studies emphasizes the virus’s limited genomic diversity across geographic regions. This recent study demonstrate that ToBRFV is primarily subject to negative selection (dN/dS < 1) (Esmaeilzadeh et al., 2023); indicating an optimized viral genome for host infection with minimal adaptive pressure for mutation. This observation aligns with our findings of host-dependent SNV patterns within the replicase proteins, suggesting a stable genome that nevertheless supports host-specific adaptations.

The high genomic similarity observed in our ToBRFV isolate from Mexico parallels findings by Van de Vossenberg et al. (2021), who reported 99.3% to 100% nucleotide identity across ToBRFV sequences from Dutch outbreaks, with up to 43 single nucleotide polymorphisms (SNPs) and limited amino acid changes. Notably, Van de Vossenberg et al. (2021) observed complete conservation in the 160 amino acid coat protein and minimal variability in the 1,621 amino acid RNA-dependent RNA polymerase (RdRp), with only up to six amino acid substitutions, underscoring the structural stability of these essential viral proteins. However, our study identifies subtle yet significant host-specific SNVs within the replicase protein, suggesting that, while ToBRFV maintains a stable genome, selective pressures may specifically influence this protein to support host-specific adaptations. This observation is further supported by Van de Vossenberg et al.’s findings of increased mutation rates in the movement protein (267 aa), which showed up to five amino acid changes, implying that certain functional domains may accommodate variability to facilitate viral spread across diverse hosts or environments. Specifically, the host-dependent variability in the methyltransferase and helicase domains identified in our study could confer adaptive flexibility, enhancing ToBRFV’s ability to establish infections across a range of hosts. Recent studies also highlight the replicase domains as a focal point for adaptive flexibility, with this region showing the highest number of synonymous and non-synonymous mutations in ToBRFV (Abrahamian et al., 2022). The elevated mutation rate may confer a selective advantage, potentially enhancing ToBRFV’s interactions with diverse host factors, enabling infection across multiple host species. The presence of host-specific SNVs within this ORF in our study supports the protein’s role in host adaptation, particularly in *S. nigrum*, *C. annuum*, and *C. lanatus*. In *C. annuum* and *C. lanatus*, SNVs identified within the RdRp domains imply modifications that could affect replication efficiency within these hosts. These findings are consistent with previous reports indicating that cucurbit hosts generally do not support ToBRFV replication under comparable experimental conditions (Zhi-yong et al., 2021; Sabra et al., 2022).

Genomic variability analysis of the Mexican isolate revealed key SNVs in domains associated with replication, movement, and host interaction. For example, within *C. annuum*, a unique thymine (T) substitution at position V2904, located in the 183 kDa replicase protein, suggests a host-specific adaptation that may enhance replication efficiency within pepper tissues. This mutation may possibly facilitate interaction with pepper-specific cellular factors, contributing to the virus’s ability to successfully establish infection in this solanaceous host. Supporting this, Ortiz et al. (2022) reported that the Mexican isolate caused the most severe symptoms in the *“tampiqueño”* chili variety among eight tested, including necrotic lesions, chlorosis, mosaic, mottling, and leaf deformation. These observations reinforce the hypothesis that host-specific selective pressures shape virus-host interactions, as *“tampiqueño”* exhibited pronounced morphological damage and reduced fruit quality compared to less affected varieties, such as habanero.

Interestingly, the three distinct SNVs found in the ToBRFV isolate from Mexico within *C. lanatus* may limit viral replication in this non-solanaceous host, suggesting that specific nucleotide variations in the replicase regions may act as genetic barriers, reducing compatibility with *C. lanatus*’ cellular machinery. Structural or functional constraints in the viral replicase could hinder optimal interactions with host-specific factors, thus impeding successful replication. Future studies could experimentally modify SNVs within the methyltransferase and helicase domains of the ToBRFV genome to match those found in isolates from compatible hosts. Such targeted mutagenesis may help elucidate the functional significance of these variants and clarify their role in shaping host specificity and restriction. In contrast, the absence of adaptive SNVs within these key domains in the Mexican isolate may underlie its inability to replicate in cucurbit hosts. This lack of host-specific modifications could hinder the virus’s interaction with cucurbit cellular machinery, thereby limiting successful infection and replication. Moreover, the distinct selective pressures imposed by cucurbit hosts—pressures that the Mexican isolate may not have historically encountered—could further explain the evolutionary divergence observed across ToBRFV populations, ultimately contributing to host specialization.

These insights are further supported by structural modeling and stability predictions. In particular, the V178I substitution—identified in *S. nigrum* but absent in *C. lanatus*—was predicted to have a destabilizing effect on the methyltransferase domain of the replicase (ΔΔG = –0.84 kcal/mol; **Supplementary Figure S7**). This suggests that specific nonsynonymous SNVs may fine-tune the structural dynamics of ToBRFV’s replicative machinery, facilitating adaptation to certain hosts. The absence of such adaptive mutations in the *C. lanatus* isolate may result in suboptimal folding or impaired interactions with host factors. Moreover, the conservation of helicase and methyltransferase domains in the Mexican isolate, which remain unchanged in *C. lanatus*, may reflect a lack of selective pressure or evolutionary time needed to adapt to this non-solanaceous host. Together, these findings point toward structural rigidity and insufficient molecular flexibility as key factors limiting host range expansion of ToBRFV. Future experimental validation using reverse genetics could help assess the functional impact of specific SNVs on replication efficiency in another host.

Overall, our results demonstrate that while ToBRFV’s genomic stability supports high infectivity in its primary host, tomato, its broader adaptability allows it to infect diverse alternative hosts. This adaptability may enable ToBRFV to establish reservoirs in non-crop plants, promoting environmental persistence and transmission potential.

### 4.3 Expanded host range of ToBRFV: observed susceptibility in native and cultivated species

Building on the observed host adaptability, experimental bioassays have revealed susceptibility to ToBRFV isolate from Mexico in *N. rustica*, *P. ixocarpa*, and *S. melongena*, further expanding the known host range of the virus. This extended host range reinforces ToBRFV’s potential to infect not only economically vital crops but also native plants, which could act as viral reservoirs within Mexico. Given the high transmissibility of ToBRFV, the infection of *P. ixocarpa* (tomatillo), a staple in Mexican agriculture, raises particular concern due to the potential for localized outbreaks that could threaten regional production. In comparison with recent studies, our findings provide a targeted view of ToBRFV host adaptability, specifically in controlled greenhouse environments and seed-borne transmission contexts. While previous research has identified *Ipomoea purpurea*, *Mirabilis jalapa*, *Clematis drummondii*, and *Solanum tuberosum* as additional natural hosts in field settings (Eichmeier et al., 2023); our study expands the known host range by confirming susceptibility in *P. ixocarpa* and *S. melongena*. These findings suggest that ToBRFV exhibits diverse adaptability across solanaceous and non-solanaceous species, which could play a significant role in viral persistence in close proximity to tomato crops.

Moreover, findings have shown that ToBRFV has naturally infected eggplant in Mexico (EPPO-192, 2019). Panno et al. (2019) could not experimentally infect this vegetable species under controlled conditions with 14 hours of light at 20° to 28°C, while using the same temperature but a photoperiod of 16 h of light, Fidan et al. (2020) detected by RT-PCR the ToBRFV in an artificial inoculated eggplants, but all were asymptomatic. Variations in temperature and in the photoperiod can undoubtedly determine whether the virus successfully infects these plants and causes symptoms.The susceptibility of *S. melongena* (eggplant) to systemic infection under specific experimental conditions aligns with findings that variations in temperature and photoperiod can influence virus infectivity (Szittya et al., 2003; Fajinmi and Fajinmi, 2010). Studies indicate that systemic infection rates and symptom expression in infected plants increase at higher temperatures, likely because these conditions promote viral replication and movement within the host (Chellappan et al., 2005; Zhang et al., 2012; Chung et al., 2015). This temperature-dependent infectivity suggests that environmental factors may play a significant role in shaping the epidemiology of ToBRFV, affecting infection dynamics across regions and climates. These findings underscore the need to consider ecological and climatic factors in managing the spread and impact of ToBRFV across different agro-ecological zones.

Our findings extend the current understanding of ToBRFV host range by demonstrating experimental susceptibility in *N. rustica*, *P. ixocarpa*, and *S. melongena*, species for which natural infections have not yet been documented. These results, obtained under controlled inoculum conditions, suggest that such species could act as incidental hosts or reservoirs under specific agricultural scenarios. This is particularly relevant for crops cultivated in proximity to infected tomato or pepper greenhouses, where mechanical transmission risk is elevated. While field surveys conducted in multiple Mexican states during 2019–2021 did not detect ToBRFV infections in these species, the capacity for experimental infection underscores the need for continued field monitoring and the implementation of preventive phytosanitary measures. Our combined approach integrating experimental infection assays with field observations provides a more comprehensive view of ToBRFV epidemiology and highlights potential vulnerabilities in diverse cropping systems.

### 4.4 Seed-borne transmission and biosecurity risks in *N. rustica*

A key challenge in studying ToBRFV seed transmission lies in the profound reproductive disruption caused by the virus in infected crop hosts, which severely limits seed availability for testing. Our observations align with this pattern: tomato and pepper plants exhibited significant sterility under infection (**Supplementary Figure S8**), consistent with previous reports. To overcome this limitation, we employed *Nicotiana rustica* as an experimental model, which remained reproductively competent despite systemic infection and allowed us to perform statistically robust seed transmission assays. While this model does not fully substitute for studies in crop species, it provides a tractable system for assessing transmission risk under controlled conditions.To further explore the adaptability and persistence of the Mexican ToBRFV isolate, we conducted seed-borne transmission assays in *N. rustica*. These assays revealed a 30% reduction in germination rates in seeds from infected plants compared to healthy controls, highlighting the virus’s capacity to impair seed viability and underscoring its potential biosecurity risks. Reduced germination has also been documented in other plant-virus interactions involving viral complexes (Marodin et al., 2019; Hemmati and McLean, 1977), although the underlying mechanisms remain poorly understood. Nonetheless, our results clearly demonstrate that viral infection can compromise seed physiological performance, warranting further investigation.

In tomato, the percentage of ToBRFV seed transmission has been recorded between 9% and 1.8% (Vargas-Mejía et al., 2023; Davino et al., 2020). The percentage of virus transmitted by the seed is different among viral species as well as among the variants of the same virus. This percentage can be modified by different factors, including mixed infections, the type of host and the stage of development during which it is infected, the severity and environmental factors that affect both the host and the performance of the virus like its mobility and multiplication in the inflorescence (Mohan et al., 2020; Cobos et al., 2019). In addition, the viral accumulation in the host and the type of transmission (vectors, contact, seed) can modify the rate of transmission of the virus (Froissart et al., 2010; Pagán, 2022).

Despite disinfection treatments, the virus persisted within seeds, posing a significant risk given the global nature of seed trade. Though infection rates in seeds and seedlings were below 1%, even minimal transmission can enable the virus to establish infection sites far from its original source, reinforcing the urgent need for biosecurity protocols (Mohan et al., 2020). The combined challenges of host adaptability, environmental resilience, and seed-borne transmission make ToBRFV a formidable pathogen with global implications for agricultural trade and plant health. Herein we detected the virus in subsamples of 150 and 100 seeds. According to Dombrovsky and Smith (2017), the threshold for detecting Tobamovirus in seeds is one infected out of 249, and 20 subsamples of 100 seeds are required to ensure a 95% likelihood of detecting the virus in minimal infestations of 0.15%. Moreover, we have observed some advantages and disadvantages that need to be analyzed to determine whether ToBRFV can be detected directly from the seed or the seedling. Seed samples can be immediately investigated, and a more significant number of seeds can be used for each sample processed during extraction, thereby increasing the likelihood of detecting the virus if a few seeds are infected. Nevertheless, non-viable virions, such as contaminants, can be found in the seed, which increases the risk of false positives. In addition, because of the seed tissue’s hardness and structural complexity, obtaining a high-quality RNA extraction is more complicated. While using seedlings makes it possible to increase the replication of the virus and thereby increase the likelihood of detection, it has been found that seedlings remain asymptomatic (with low viral load) and do not express symptoms until handled in intensive production. Meanwhile, analyzing only seedlings without the seed coat eliminates the risk of false positives. The seedling tissue is easier to process, increasing the quality of the extracted RNA. Nonetheless, fewer can be processed since the plant material weighs more in seedlings. There needs to be more information about how many seedlings per subsample need to be processed to detect tobamoviruses.

In general, surface disinfection of seeds with 1-3% sodium hypochlorite (NaOCl) has been shown to be effective against viruses (Herrera-Vásquez et al., 2009), and it has been corroborated that 2.5% NaOCl for 15 minutes was able to inactivate ToBRFV (Davino et al., 2020). Sodium hypochlorite effectively disinfected seed surfaces, with no ToBRFV detected via RT-PCR, supporting its use in seed certification protocols. However, the virus’s persistence within seed tissues underscores the need for molecular diagnostics to detect internal infections and prevent the spread of asymptomatic, infected seeds in international markets.

Our findings highlight the need for an integrated approach to manage ToBRFV’s host versatility, seed-borne transmission, and environmental resilience. Host-specific adaptations in replicase proteins emphasize the importance of developing resistant cultivars tailored to regional strains. The ToBRFV isolate from Mexico demonstrates the virus’s ability to maintain genomic stability while adapting to diverse hosts and environments, reinforcing its status as a significant global pathogen. This study provides valuable insights into ToBRFV’s adaptability and supports practical biosecurity and management strategies to mitigate its impact on global agriculture.

## 5 Conclusions

This study provides insights into the genetic landscape and host interactions of a Mexican ToBRFV isolate. Genomic analyses revealed variability concentrated in replicase regions, with strong sequence similarity to strains from diverse geographic regions. These findings support the hypothesis that international trade, particularly of seeds and plant material, may play a role in the global dissemination of ToBRFV, although direct transmission pathways remain to be elucidated. The observed susceptibility of native plant species and the apparent temperature-dependent infectivity in eggplant highlight the importance of considering ecological and environmental factors in future surveillance efforts. Overall, our results underscore the relevance of continuous monitoring, the development of resistant cultivars, and the implementation of coordinated biosecurity strategies to manage the potential spread and impact of ToBRFV in both local and international agricultural systems.

## Data Availability

The datasets presented in this study can be found in online repositories. The names of the repository/repositories and accession number(s) can be found below: https://www.ncbi.nlm.nih.gov/genbank/, BankIt2895689. https://github.com/kap8416/TOBRFV-Genome-Analyses/tree/main.
