# Supplementary material for: Genomic insights into host-associated variants and transmission features of a ToBRFV isolate from Mexico
Source: Front Plant Sci. 2025 Aug 15;16:1580000. doi: 10.3389/fpls.2025.1580000 (PMC12394481; doi:10.3389/fpls.2025.1580000)
Supplement: Supplementary file 1 [file DataSheet1.docx]

***Supplementary Material***

# Supplementary Data

**Table S1.Parameters of Mexican ToBRFV isolate sequencing**

|  | **Library Name** | **Total Read Bases** | **Total Reads** | **GC (%)** | **Q20 (%)** | **Q30 (%)** |
| --- | --- | --- | --- | --- | --- | --- |
| Raw data stats | RNAtotal_1 | 7,570,633,546 | 50,136,646 | 50.15 | 97.03 | 92.37 |
| Filtered data stats | RNAtotal_1_trimmed | 6,083,869,459 | 44,518,584 | 50.35 | 98.93 | 95.61 |
|  | RNAtotal_1_reduced | 13,573,155 | 99,338 | 50.34 | 98.92 | 95.57 |

**Table S2. Overall mapping stats**

| **Library Name** | **Total reads** | **Mapped reads** | **Coverage (%)** | **Depth** | **Ins. Size (Std.)** |
| --- | --- | --- | --- | --- | --- |
| RNAtotal_1 | 99338 | 31201 (31.41%) | 100.00 | 607.16 | 171.62 (59.66) |
| Total | 99338 | 31201 (31.41%) | 100.00 | 607.16 | - |

**Table S3. Base contents of contigs**

| **Num of A** | **Num of T** | **Num of G** | **Num of C** | **Num of N** | **GC contents** |
| --- | --- | --- | --- | --- | --- |
| 1938 | 1789 | 1467 | 1181 |  | 41.54% |
| **Contig name** | **Length** | **GC (%)** | **Circular** | **Contig name** | **Length** |
| Contig1 | 6375 | 41.54 | NO | Contig1 | 6375 |
| Total | 6375 | 41.54 |  | Total | 6375 |

# Supplementary Figures

1. B)


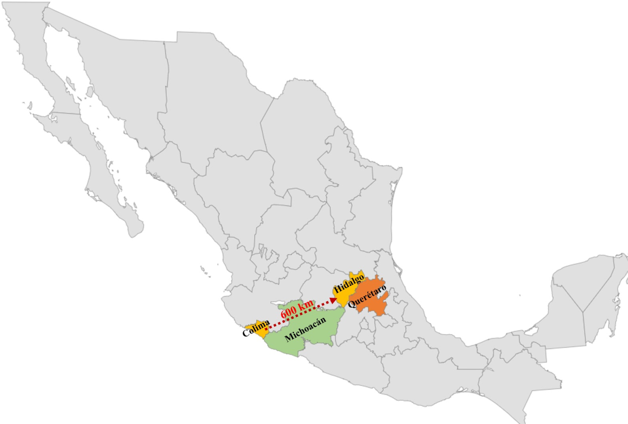

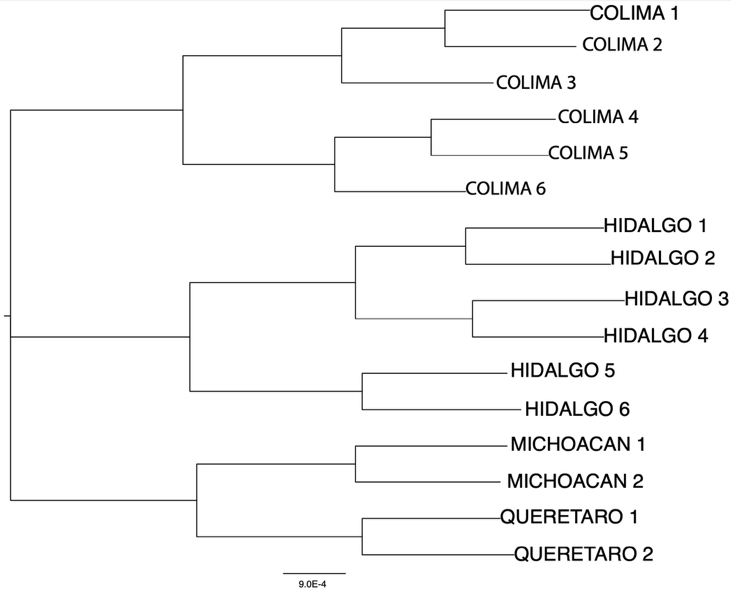


**Supplementary Figure S1.** **Geographic Distribution and Phylogenetic Relatedness of Mexican ToBRFV Isolates.** (A)Geographic distribution of ToBRFV field sampling sites across four Mexican states: Michoacán, Colima, Querétaro, and Hidalgo. Colored regions indicate sampling locations. Notably, full-genome sequencing revealed that the viral isolates from Colima and Hidalgo were genetically identical, despite being collected over 600 km apart. (B) Phylogenetic tree of *ToBRFV* isolates from four Mexican states. Sequences from reverse reads were aligned, and a neighbor-joining tree was built. The clustering shows no clear phylogeographic structure, suggesting high sequence similarity among isolates regardless of geographic origin.


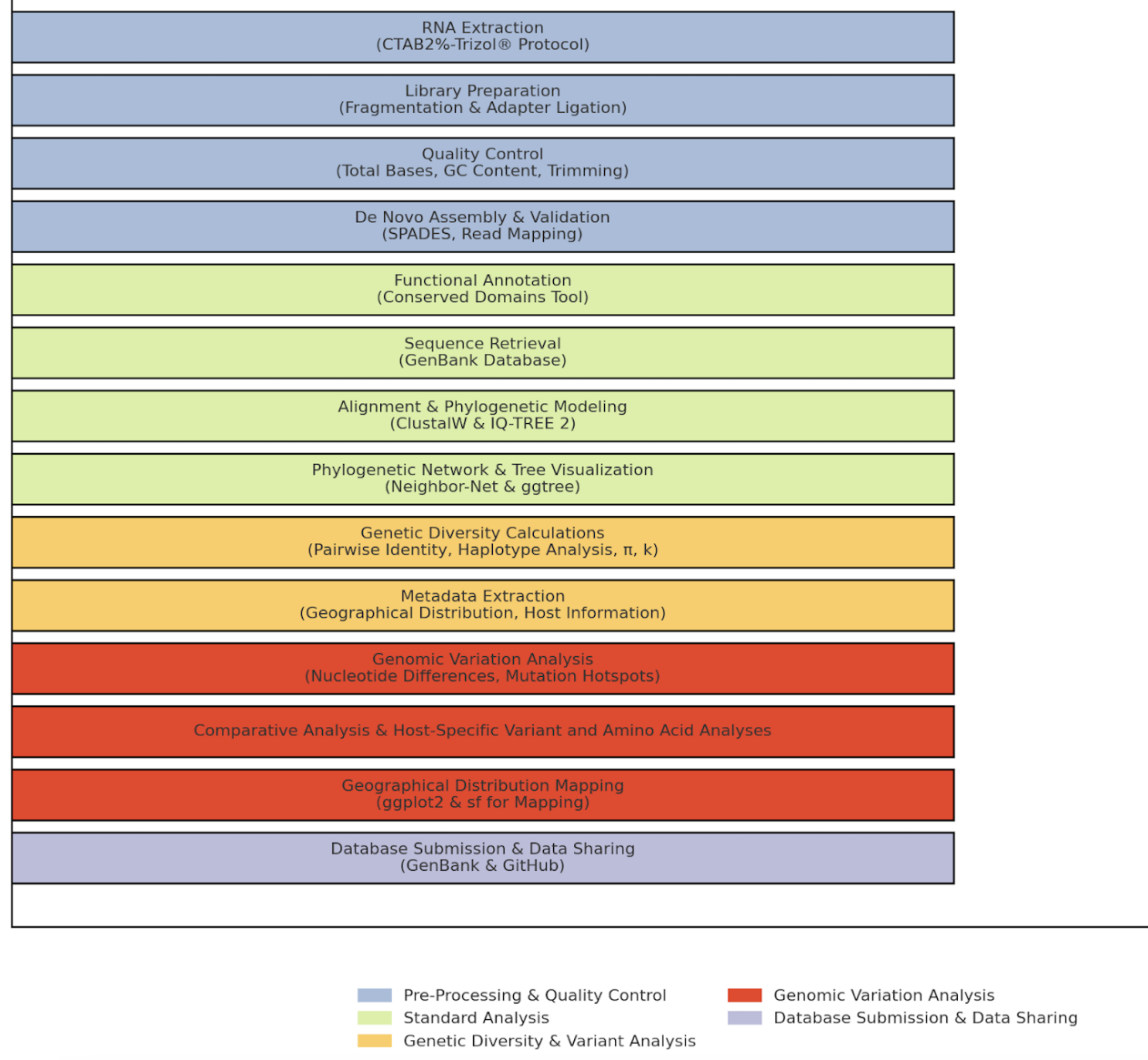


**Supplementary Figure S2.** Workflow for the *in silico* analysis of the ToBRFV Mexican isolate.
The workflow includes the following key steps, categorized by function: *Pre-Processing & Quality Control (blue)*: Includes RNA extraction, library preparation, quality control, and de novo assembly and validation. *Standard Analysis (green***)**: Functional annotation, sequence retrieval; full genome sequences of 100 ToBRFV isolates are retrieved from GenBank, alignment and phylogenetic modeling; sequences are aligned with ClustalW, and IQ-TREE2 is used to perform phylogenetic modeling with 1000 bootstrap replicates, and phylogenetic network visualization; Neighbor-Net and ggtree in R are used to visualize the phylogenetic relationships among isolates. *Genetic Diversity & Variant Analysis (orange)*: Genetic diversity calculations  (pairwise identity, haplotype analysis, π, k), metadata extraction geographical and host information is extracted for each isolate, and geographical distribution mapping. *Genomic Variation Analysis (red):* Nucleotide differences, mutation hotspot identification, and comparative host-specific variant and amino acid analyses. *Database Submission & Data Sharing (purple)*: Final step for submission and sharing of annotated data with GenBank and GitHub.


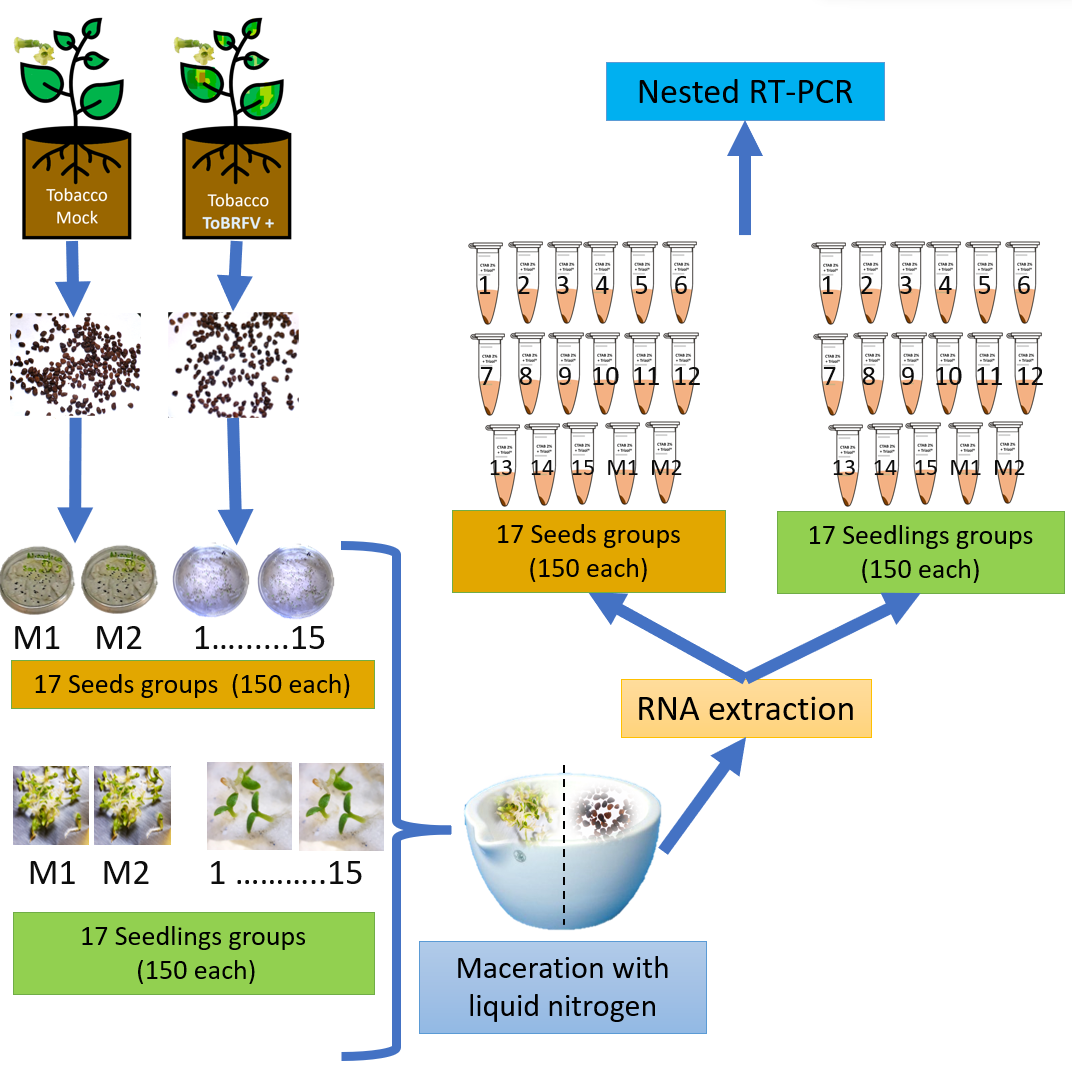


**Supplementary Figure S3.** Schematic detailing the procedure for determining the percentage of infection in seeds and seedlings. Groups M1 and M2 symbolize mock-inoculated controls. The numbers 1-15 represent individual groups of seeds and seedlings examined.


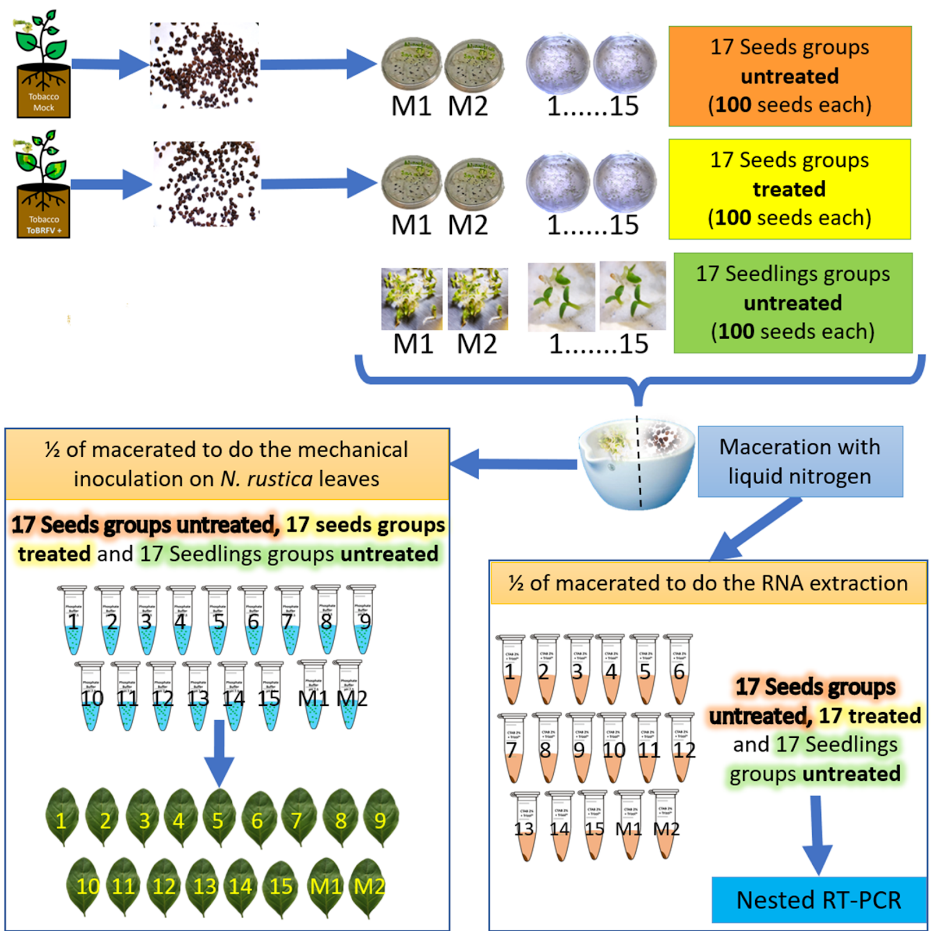


**Supplementary Figure S4.** Flowchart illustrating the evaluation of viral particle infectivity in seeds. M1 and M2 denote the mock-inoculated controls. Groups 1-15 signify distinct batches of seeds and seedlings. Notably, all seed groups underwent a treatment with a 3% sodium hypochlorite solution for a span of 3 minutes.


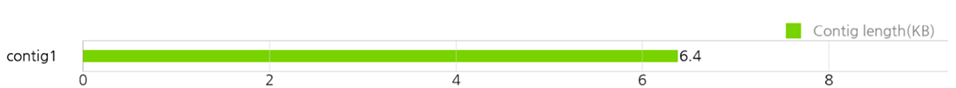


 
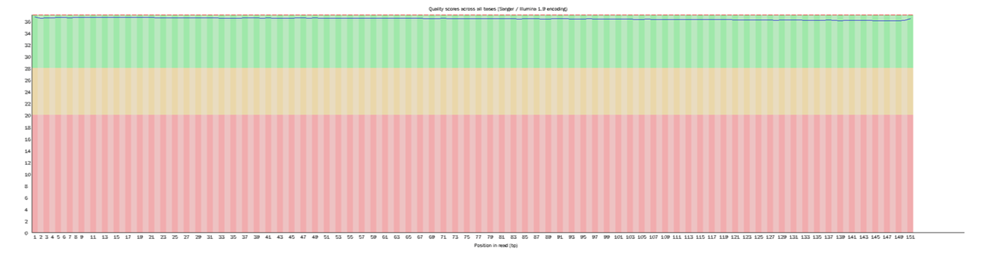


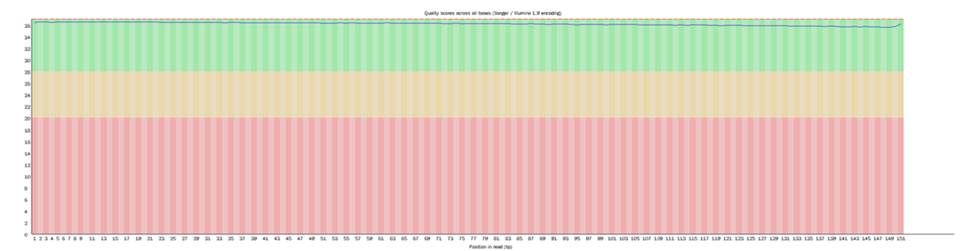


**Supplementary Figure S5.**     Figure of longest contigs and base quality of the ToBRFV sample (up: read; 1down: read2) at each cycle after filtering


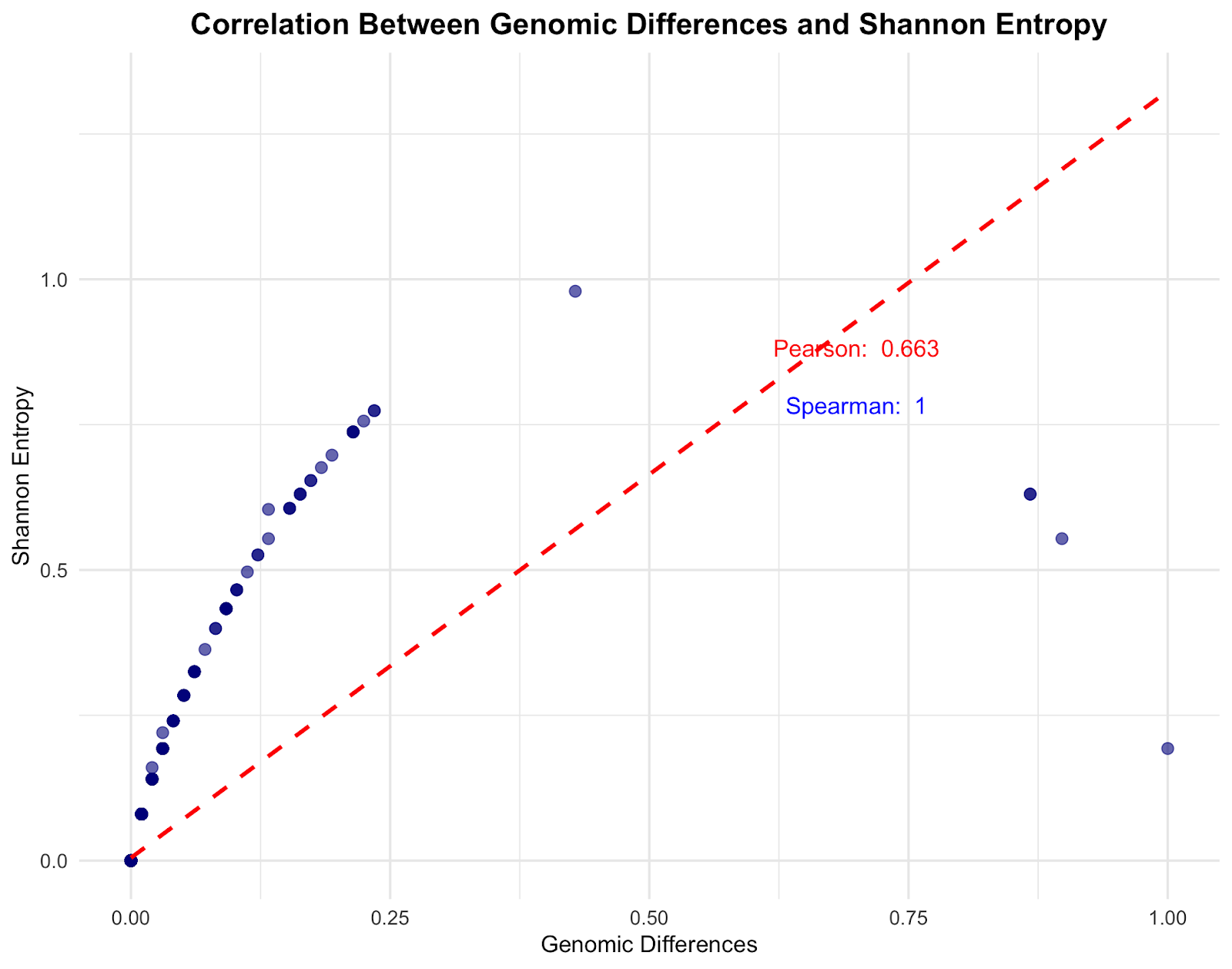


## Supplementary Figure S6. Correlation Between Genomic Differences and Shannon Entropy. Scatter plot showing the relationship between genomic differences (x-axis) and Shannon entropy (y-axis) for positions in the *ToBRFV* genome. The red dashed line represents the Pearson correlation coefficient (r=0.663), indicating a moderate linear relationship. The Spearman correlation coefficient (r= 0.999) highlights an almost perfect monotonic relationship, suggesting that positions with higher differences consistently show greater sequence variability.


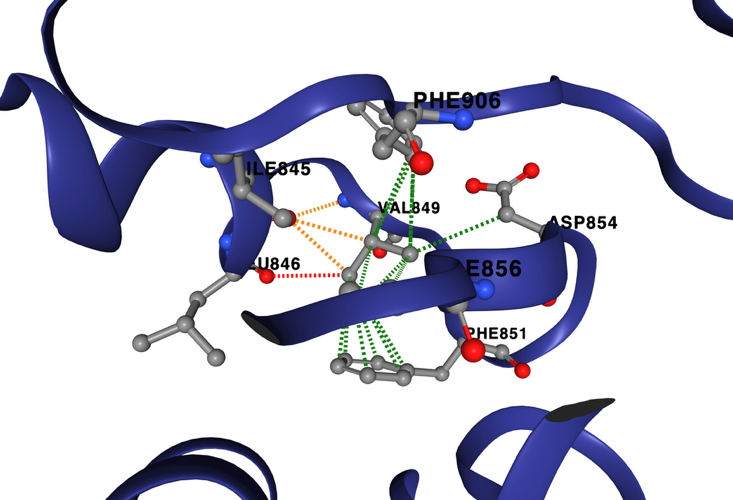

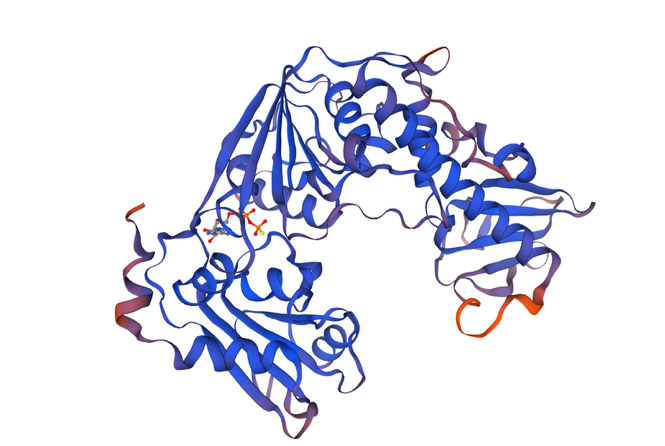


**Supplementary Figure S7.** **Predicted structural impact of the V1781 mutation in the methyltransferase domain of the ToBRFV replicase as visualized by DynaMut2.** The mutation was modeled at position 849 of the Swiss Model-predicted structure. DynaMut2 predicts a destabilizing AAG of -0.84 kcal/mol. Regions affected by the mutation are highlighted, suggesting local flexibility changes in the methyltransferase domain.


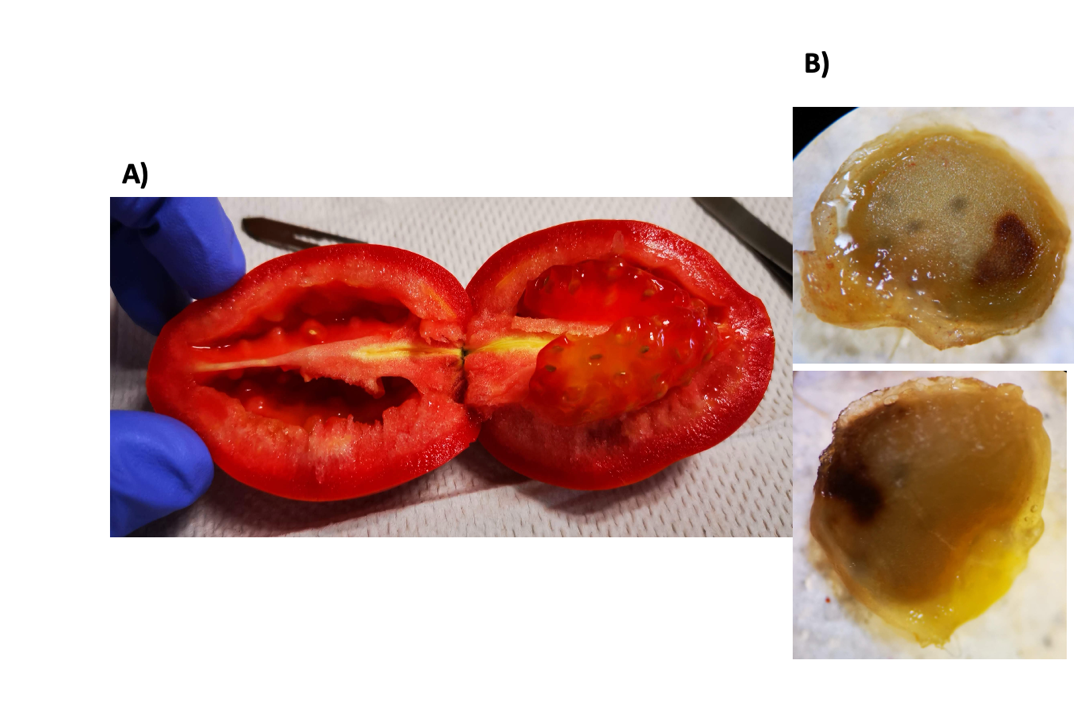


## Supplementary Figure S8. Internal fruit and seed symptoms observed in ToBRFV-infected tomato tissues. (A) Cross-section of a tomato fruit infected with ToBRFV displaying uneven ripening and degradation of the locular gel. (B) Seeds extracted from the same infected fruit exhibit internal necrosis and brown discoloration, consistent with virus-associated physiological damage. These findings highlight the impact of ToBRFV infection on both fruit integrity and seed quality.
